# Global Evidence on Monitoring Human Pesticide Exposure

**DOI:** 10.3390/jox15060187

**Published:** 2025-11-07

**Authors:** Tatiane Renata Fagundes, Carolina Coradi, Beatriz Geovana Leite Vacario, Juliana Maria Bitencourt de Morais Valentim, Carolina Panis

**Affiliations:** 1 Laboratory of Tumor Biology, Western Paraná State University, Francisco Beltrão 85605-010, Parana, Brazil; tatiane.fagundes@uenp.edu.br (T.R.F.); carolina.coradi@uel.br (C.C.); juliana.mbitencourt@uel.br (J.M.B.d.M.V.); 2 Laboratory of Mutagenesis and Oncogenetics, State University of Londrina, Londrina 86057-970, Paraná, Brazil; beatriz.geovana@uel.br

**Keywords:** pesticide, environmental pollutants, biological monitoring

## Abstract

This study analyzes global data on human exposure to pesticides, focusing on glyphosate, POPs, carbamates, and organophosphates, which are among the most widely used in agricultural and urban environments, providing an overview of global human contamination by these substances. Current research has increasingly focused on the unintended consequences of pesticide use, including food, water, and soil contamination, biodiversity loss (especially beneficial insects such as pollinators), and the growing evidence of adverse impacts on human health (neurological, reproductive, endocrine, and carcinogenic effects). Therefore, we compiled information from several existing studies that evaluated pesticide residues in human biological samples, specifically urine, blood, and breast milk, to assess the extent of exposure. The analysis takes a global perspective, highlighting the importance of monitoring exposure in countries that demonstrate exceptionally high pesticide use (in terms of absolute volume), such as Brazil, the United States, and China, which are among the largest global consumers. The data cover both contemporary pesticides, whose consumption is driven by intensive agriculture in these and other countries, and persistent legacy compounds (POPs) that continue to circulate in nature and accumulate in the human body decades after their ban in many countries. Globally, there is a wide disparity in global regulations, and many developing countries continue to use pesticides that have been banned or severely restricted in more developed nations. Finally, it provides a critical overview of global data on human pesticide contamination. The data reinforce the critical importance of establishing preventive initiatives and strengthening surveillance and monitoring systems to detect and control pesticide residues in human populations globally, ultimately aiming to mitigate the harms of chronic pesticide exposure to human health and well-being.

## 1. Introduction

The widespread use of pesticides in global agriculture has been a cornerstone of ensuring food security and increasing agricultural productivity [1]. However, this intensive reliance raises significant concerns about the potential risks to human and environmental health, given the persistence and toxicity of many of these compounds.

This exposure occurs through occupational, environmental, and incidental pathways, including unintentional contact, such as spray drift or food and water contamination, and has been linked to a variety of health problems, including cancer, diabetes, and neurodegenerative diseases. Furthermore, the presence of these substances may be associated with adverse maternal and child health outcomes, such as congenital anomalies and reproductive dysfunction [1,2].

Despite historical limitations, such as complex and costly analytical procedures and the need for specialized laboratories, there has been significant progress in the biomonitoring of human pesticide exposure. Validated methods for common pesticide metabolites are routinely applied, and international initiatives have supported standardization and quality assurance efforts. At the same time, several regulatory agencies (e.g., European Food Safety Authority (EFSA), European Chemicals Agency (ECHA), and United States Environmental Protection Agency (US EPA)) have published reference values for selected compounds, contributing to better interpretation of biomonitoring data. Although health-based reference values, such as the Acceptable Daily Intake (ADI), exist, these values vary significantly between countries, making standardization of reference values in human matrices difficult. Consequently, biological indices of exposure, defined as concentrations of pesticides or their metabolites in human samples, such as urine or blood, indicative of exposure levels, are still lacking or inconsistent for several compounds [3].

Pesticide regulations vary significantly across countries. Some impose strict standards, while others adopt more lenient policies, influenced by numerous factors, including agricultural practices, pest prevalence, and regional environmental conditions, which increase population exposure in areas with more lenient regulations. In the early 2000s, pesticide application rates were highest in Asia and the Americas, averaging 2.92 and 2.42 kg/ha, respectively. In 2018, these rates increased to 3.69 kg/ha in Asia and 3.54 kg/ha in the Americas. In contrast, pesticide use in Europe, Africa, and Oceania during 2018 was considerably lower, at 1.67, 0.39, and 2.07 kg/ha, respectively [4]. Therefore, distinct patterns of exposure may emerge among populations around the world.

According to the 2022 Food and Agriculture Organization of the United Nations (FAO) report, the Americas led global pesticide use, applying over 1 million tons, with an average intake of 1.17 kg per person [5]. Europe followed with an average of 0.64 kg/person, while Africa recorded lower use than the others, with an intake per person of 0.11 kg/person. In terms of the absolute amount applied in 2020, the top three countries were the United States (0.408 million tons), Brazil (0.377 million tons), and China (0.273 million tons) [6], countries with the most significant monitoring data due to their high rates of use of these compounds. We also present data from many European Union countries, which historically have stricter legislation on the usual limits for these compounds in food and water, and compare the regulatory differences between them.

Most pesticide biomonitoring studies in humans are conducted in high-income countries, such as those in the European Union and North America, creating a significant lack of data in low- and middle-income countries, where agricultural practices, the use of personal protective equipment, and regulations may be less stringent, exposing populations to greater and poorly documented risks, limiting the creation of public policies to address this problem. Data on pesticide exposure and its health effects are from studies that are cross-sectional and not longitudinal, limiting the understanding of the effects of long-term exposure and hindering the creation of standardized global biomonitoring programs that allow for comparison of data across countries and populations. This also impedes the creation of a globally comparable database to assess exposure trends over time and identify more vulnerable populations.

Against this backdrop, it becomes evident that human exposure to pesticides results not only from their intensive use but also from regulatory weaknesses, the insufficiency of consistent monitoring programs, and the absence of effective environmental remediation strategies. Therefore, this review is guided by the hypothesis that legislative permissiveness, the lack of systematic biomonitoring, and the irreversibility of environmental and biological contamination act together to intensify risks to human health and ecological balance. These shortcomings are further aggravated by socioeconomic inequalities, which leave groups such as rural workers, pregnant women, and children disproportionately vulnerable, particularly in countries with more lenient regulations, in contrast to the more precautionary framework.

In this context, this review presents a global overview of human pesticide contamination based on biomonitoring data from biological samples of blood, urine, and breast milk, focusing on current and past pesticides, including glyphosate and its metabolite AMPA, DDT, and its organophosphate and carbamate derivatives DDD and DDE. It provides an overview of the global landscape of environmental contamination and its currently documented effects on human health. Reviewing this topic is crucial to synthesizing existing evidence and highlighting its gaps, providing a solid basis for public health decision-making and the formulation of more effective regulatory policies. The lack of data in certain regions and the inability to assess cumulative exposure hinder the definition of safety limits and the implementation of risk mitigation strategies globally.

To make the structure of the article clearer, the authors have provided an outline of the manuscript, which is included in the Supplementary Materials.

## 2. Pesticide Levels in Human Samples: Primary Matrices, Methods, and Target Analytes

Blood and urine are the most commonly used biological samples for pesticide detection. Blood is considered an ideal matrix for detecting many chemical compounds, as it circulates throughout the body, promoting exchanges between tissues and organs. Thus, whole blood or serum and plasma are important to assess internal exposure to persistent pesticides, such as organochlorine insecticides, which have long biological half-lives and can be measured as the parent compound or its metabolites [7].

For pesticides with a short half-life in the human body and therefore found in low concentrations in the blood, urine is the matrix of choice, especially for the evaluation of non-persistent pesticides such as pyrethroids, organophosphate insecticides, or different classes of herbicides, such as glyphosate, which, due to its rapid metabolism, limits its detection in blood samples [8]. The compounds found in urine indicate recent exposure, as non-persistent pesticides are metabolized and eliminated from the body within a few days.

Non-invasive alternative matrices for monitoring pesticides, such as hair, meconium or breast milk, have attracted increasing interest in recent years due to their ease of acquisition. Human hair is an easy-to-collect, -transport, and -store sample that is stable and provides information on short- and long-term exposures, but it is difficult to differentiate between endogenous deposition, diffusion from blood capillaries, and exogenous environmental deposition. Several organochlorine and organophosphate insecticides have been found in hair samples, mainly by analysis of their parent compound [9].

Compounds that have lipophilic bioaccumulation characteristics are excreted in breast milk, a sample that can be used to monitor maternal and infant exposure to lipophilic organochlorine pesticides and other persistent organic pollutants (POPs). Comparison of the levels found should be adjusted according to the lipid content, since the amount of lipids in milk varies. Milk analysis requires careful preparation, with volumes between 1 and 5 mL and removal of proteins and fats [10].

Meconium is considered an appropriate matrix to measure prenatal pesticide exposure, since these compounds can accumulate in meconium from the third month of gestation until birth. This analysis provides a long-term fetal exposure profile and can be collected in large quantities [11].

There are many quantitative methods for detecting pesticides in biological samples. Sample preparation involves multiple steps that remove interfering substances or hydrolyze conjugated forms of the compounds of interest (pre-analytical step). In some cases, pre-treatment consists of a simple dilution of the sample with water or formic acid to reduce the matrix effect and matrix variability between samples, as is done with urine [10,11]. Blood or breast milk samples need to undergo precipitation processes and protein denaturation, and the compounds used for this purpose include formic acid and solvents such as n-propanol, isopropanol, methanol or acetonitrile, which increase the extraction efficiency of lipophilic pesticides and avoid clogging of solid phase extraction [10,12,13,14].

To protect public health, the European Commission (EC) established Maximum Residue Limits (MRLs) for pesticides in food, measured in µg/kg or mg/kg [15]. MRLs vary depending on the matrix analyzed, the specific environmental occurrence, and the toxicological profile of the pesticide. A general MRL of 10 µg/kg is established when an MRL value is not specified [16]. Therefore, monitoring pesticide residues in food becomes essential to comply with these [17].

The wide variety of pesticides legally approved for use requires multi-residue analytical methods for their determination. However, determining low detection limits in accordance with the established MRLs and the complexity of some biological matrices, the different chemical classes to which these compounds belong, complicate this analysis [18].

The analysis of pesticides in human matrices still uses classical extraction and purification strategies, such as solid-phase extraction (SPE), liquid–liquid extraction (LLE), solid–liquid extraction (SLE), and solid-phase microextraction (SPME). Liquid–liquid extraction is a routine technique used for many years, but it requires a long time and solvent use and has been replaced by solid-phase extraction recently. Solid-phase extraction is an important approach for extracting and purifying pesticides from urine and milk [19,20]. For urine, several solvents are used in the extraction and purification of pesticide metabolites from urine. Oasis HLB, for example, is especially recommended for specific metabolites of organophosphates, pyrethroids and herbicides, while Strata XC (mixed-mode reversed phase/strong cation exchange) is more suitable for atrazine metabolites [21,22].

QuEChERS is a proprietary sample preparation method that has become extremely popular and is often considered an alternative or evolution for the analysis of pesticide residues in complex matrices. Its simplicity and effectiveness have allowed laboratories around the world to adopt similar protocols, which addresses the issue of the lack of standardized methods in different countries. QuEChERS (acronym for quick, easy, cheap, effective, robust, and safe)-based methods consist of analyte extraction by liquid partition using a small volume of acetonitrile, subsequent collection of the organic fraction after a salting-out step (promoting equilibrium between an aqueous and an organic layer), and cleanup of the organic extract using dispersive solid-phase extraction (d-SPE) [17]. The versatility of this approach allows the use of different buffered salts for the salting-out step, depending on the matrix and pesticide properties. This method is widely applicable in biological matrices with good extraction efficiency for a large number of organic compounds, allowing the selective extraction of multi-residue pesticides with a significant reduction in interferents in the sample and achieving high extraction efficiencies for pesticides from different chemical groups [23].

The choice of instrumental techniques for separating, detecting, and quantifying pesticides is crucial and depends on the physicochemical properties and concentrations of the target compounds. Gas chromatography–mass spectrometry (GC-MS) and high-performance liquid chromatography–tandem mass spectrometry (HPLC-MS/MS) are commonly used for pesticide detection. GC-MS is well-suited for nonpolar and volatile compounds, such as organochlorines (OC), pyrethroids (PYR), and organophosphates (OPs), or for more polar compounds, such as dialkyl phosphates (DAPs), when a prior derivatization step is used. In contrast, HPLC-MS/MS is preferable for more polar, less volatile, or thermally labile compounds, including metabolites of OPs and PYR, triazine herbicide metabolites, phenoxy acid herbicides, and neonicotinoid metabolites.

Conventional LC-MS/MS analysis determines a list of known analytes through the use of reference standards. LC-HRMS offers an alternative for broad screening of hundreds of polar contaminants in full-scan mode, enabling the detection of both known and unknown non-target compounds with high sensitivity and selectivity. However, in the absence of reference standards, quantification is generally limited to a semi-quantitative level [24,25].

Persistent pesticides are analyzed together with other POPs (polychlorinated biphenyls (PCBs) and dioxins), covered by the Stockholm Convention. The effectiveness of this treaty has led to a decrease in the concentrations of persistent pollutants in environmental and biological matrices, and more sensitive instrumental methods are needed to reliably quantify the amounts of persistent pollutants in mixtures of compounds [26]. The GC-MS method is the routine method for quantification of persistent pesticides and other POPs in various environmental and biological matrices [27].

The literature demonstrates that various regions and populations across Latin America, European Union countries, North America, Asia, and some African countries have experienced harmful health effects due to prolonged occupational or dietary exposure to pesticides, even at low doses, reinforcing that pesticide contamination is a global phenomenon. Additionally, these substances are known to quickly disperse through the atmosphere and infiltrate soil, contaminating groundwater sources such as water tables and aquifers, rendering them unsafe for human consumption [28].

Much research has been carried out mainly in the ten countries that currently use the highest volumes of pesticides worldwide: China (1806 million kg), the United States (386 million kg), Argentina (265 million kg), Thailand (87 million kg), Brazil (76 million kg), Italy (63 million kg), France (62 million kg), Canada (54 million kg), Japan (52 million kg), and India (40 million kg) [29,30]. The widespread and often uncontrolled use of pesticides in these countries is largely driven by the demand to maximize crop productivity, contain pests, and prevent crop diseases, improving harvest efficiency and ensuring significant economic returns for the agricultural sector. However, this dependence harms both the biosystem and rural communities and consumers [29].

Globally, the most widely used pesticides in countries where agriculture is the main economic activity include trifluralin, 2-methyl-4-chlorophenoxyacetic acid, glyphosate, 2,4-D, mancozeb, aldicarb, atrazine, chlorthalonil, diazinon, dicamba, diuron, malathion, metolachlor, and chlorpyrifos. Among these and other pesticides, many are banned in certain European Union countries but remain widely used in developing nations; these pesticides include chlorpyrifos, endosulfan, cypermethrin, atrazine, paraquat, glyphosate (both dimethylammonium and monoammonium salts), tebuthiuron, lambda-cyhalothrin, imidacloprid, dichlorvos, emamectin, and mancozeb [5,31,32].

While banned in countries in the Northern Hemisphere due to their harmful effects on the human body and ecosystems, they continue to be widely used in other countries in the Southern Hemisphere, where regulations are more lenient. Consequently, countries with stricter domestic policies may still expose their populations to banned pesticides through the import of products such as coffee, orange juice, and soybeans, which have not been adequately evaluated and come from countries with more lenient regulations [29]. Maggi and Cols (2021) [33] analyzed three key variables to assess and correlate pesticide use in different countries with potential risks to human health, including pesticide toxicity, gross population exposure based on geographic distribution, and human intake through air inhalation and drinking water contamination. This approach highlights how the type and scale of pesticide use, combined with local exposure pathways, contribute to varying levels of health risk across regions. The assessment revealed that critical regions common to all three variables were East Asia, mainly China, and South America, specifically Brazil. The study also highlighted that European Union countries, despite high pesticide application rates, exhibit lower average risk levels to their populations because of severe prohibitions and laws governing the application of these substances.

Therefore, there is a clear need for globally harmonized and stricter regulations on pesticide bans and maximum residue limits that are not regionally flexible. Such measures would better protect public health from risks such as cancer, endocrine disruption, neurological disorders, and environmental harm.

Among the various types of pesticides currently in use, glyphosate, POPs, carbamates, and organophosphates rank among the most utilized across agricultural and urban environments. These compounds are increasingly scrutinized due to their potential to compromise human health and disturb ecological systems. Proper assessment of their environmental fate and effects requires a thorough understanding of their physicochemical properties, as these determine how they interact with soil, water, air, and living organisms, as well as their degradation dynamics and persistence within ecosystems [34].

In the following sections, we will examine the principal pesticides used globally, the concentrations found in biological matrices, and the analytical techniques applied to measure them.

### 2.1. Glyphosate-AMPA

#### 2.1.1. Chemical Composition and General Properties

**Figure 1:**
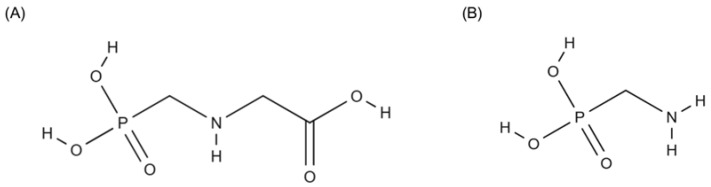
Chemical structure of glyphosate (**A**) and AMPA (**B**) [36].

Glyphosate (N-phosphonomethyl-glycine) (Figure 1) is an organophosphorus compound used as a broad-spectrum, non-selective herbicide, widely applied in agriculture for weed control. Its mechanism of action is based on the inhibition of the enzyme 5-enolpyruvylshikimate-3-phosphate synthase (EPSPS), which is involved in the shikimate pathway—essential for the biosynthesis of aromatic amino acids (phenylalanine, tryptophan, and tyrosine) in plants [35].

It was in the 1970s that glyphosate was introduced to the market by the company Monsanto, under the trade name Roundup. Since then, it has become one of the most widely used pesticides worldwide. With the introduction of genetically modified crops resistant to glyphosate, its use has intensified [37].

The main method of glyphosate application in large agricultural areas is through aerial or ground spraying, which facilitates its dispersion through the air to non-target areas. After application, glyphosate can contaminate the environment through spray drift, surface runoff and leaching into groundwater. Residues of glyphosate and its metabolite, aminomethylphosphonic acid (AMPA), have already been detected in rivers, lakes, groundwater, and even in drinking water [38,39]. It is a compound moderately soluble in water (12 g/L at 25 °C) and exhibits adsorption to soil particles. Being stable to chemical and photochemical decomposition, its degradation depends on both biotic and abiotic factors and is mediated by microorganisms such as species of the genera *Pseudomonas* and *Achromobacter*, which produce AMPA and glyoxylic acid [40].

Currently, glyphosate is one of the main environmental contaminants and can affect non-target organisms such as soil microorganisms, amphibians, and insects. Reports indicate that its presence alters soil microbiota and interferes with nitrogen fixation in legumes, impacting soil fertility [41]. In aquatic environments, glyphosate can be toxic to algae, fish, and amphibians, especially in formulations that contain surfactants such as POEA (polyethoxylated tallow amine) [42].

The application of glyphosate on food crops leads to detectable residues in foods consumed by the general population. Studies have identified glyphosate and AMPA in various foods such as cereals, legumes, fruits, and vegetables, raising concerns about the effects of chronic ingestion of these residues [43].

In 2015, glyphosate was classified as probably carcinogenic to humans (Group 2A) by the International Agency for Research on Cancer (IARC), based on evidence of genotoxicity and the occurrence of tumors in laboratory animals under high levels of exposure [44]. However, this classification remains the subject of international debate by regulatory agencies, which allege insufficient evidence of carcinogenicity in humans under typical exposure conditions. Human exposure occurs mainly through occupational routes, ingestion of contaminated food and water. Rural workers involved in the preparation and application of pesticides are considered the most exposed individuals; however, women who do not work directly in the fields but are responsible for decontaminating clothing and personal protective equipment of their male partners who work in the fields show high levels of glyphosate contamination, mainly due to dermal exposure [45].

Chronic exposure to glyphosate has been associated with various conditions, such as alterations in immune system cells [46], breast cancer [45], follicular lymphoma [47], neurological disorders [48] and infertility [49].

#### 2.1.2. Regulatory Limits in Different Countries and Health Effects in Humans

Regulation of the maximum allowable levels of glyphosate in drinking water varies significantly between countries and international organizations, reflecting different risk assessment approaches as well as the influence of economic policies and regulatory precautions. The European Union (EU) adopts one of the strictest standards worldwide, setting the limit at 0.1 µg/L for any individual pesticide, including glyphosate, in its Directive 98/83/EC on drinking water quality [50]. In contrast, countries such as the United States and Brazil have considerably more permissive limits. The U.S. Environmental Protection Agency (EPA) sets a Maximum Contaminant Level (MCL) of 700 µg/L [51]. In Brazil, the Ministry of Health, through Ordinance GM/MS No. 888/2021, establishes a limit of 500 µg/L [52].

Other countries also adopt different limits. In Canada, the maximum allowable value is 280 µg/L, according to Health Canada guidelines [53]. Australia is even more permissive, allowing up to 1000 µg/L, based on criteria established by the National Health and Medical Research Council (NHMRC) [54]. The World Health Organization (WHO), in its most recent provisional guideline, proposes a safety limit of 900 µg/L for glyphosate in drinking water [55].

This wide disparity in regulatory limits demonstrates the lack of international scientific consensus regarding the safety of glyphosate at residual levels, which hinders global risk comparisons and compromises coordinated strategies for environmental monitoring and public health protection.

Concerning human exposure, Table 1 presents the levels of glyphosate and AMPA detected in urine samples from individuals from different countries.

The compilation of biomonitoring studies demonstrates that human exposure to glyphosate and AMPA is a global phenomenon, with residues detected in urine samples from children, adolescents, adults, and pregnant women in both rural and urban settings. Detection levels vary among studies, reflecting differences in the intensity of herbicide use, dietary habits, proximity to agricultural areas, application methods, regulatory policies, and the analytical sensitivity of the laboratory methods employed.

The detection of glyphosate and AMPA in urine samples from various EU countries highlights widespread environmental exposure to this herbicide, especially considering the 0.1 µg/L limit for pesticides in drinking water. Available data show that both adults and children are exposed to glyphosate, with detectable levels found in rural and urban populations. Studies conducted in Belgium [56], France [62] and Germany [64], indicate higher contamination in children and adolescents, reflecting the greater susceptibility of these groups. In France, detectable levels of AMPA were higher in children than in adults. In Belgium, greater contamination was associated with proximity to agricultural areas, underscoring the importance of geographic location as a variable in environmental exposure. 

The association between AMPA and oxidative stress markers observed in Cyprus [60] reinforces concerns about the biological effects of pesticide exposure, especially in developing populations such as children. This finding is consistent with previous literature indicating that glyphosate can induce oxidative and genotoxic damage in experimental models [93,94,95].

Furthermore, dietary patterns have proven to be an important exposure factor in adult populations. In Germany [65], positive correlations were identified between urinary glyphosate excretion and legume consumption, and between AMPA excretion and mushroom consumption. In Spain [73], a relationship was observed between the presence of urinary glyphosate in infants and recent consumption of fruits and eggs.

In rural workers, such as vineyard workers in Italy [67], detected levels were substantially higher (median of 2.30 µg/L), reflecting occupational absorption following product application. This exposure is concerning given that several studies suggest that occupational exposure to glyphosate may be linked to adverse health effects, including endocrine disruption and an increased risk of certain cancers, such as non-Hodgkin lymphoma [96].

Even in countries with lower reported agricultural use, such as Ireland [66] and Portugal [71], measurable concentrations of glyphosate and AMPA were detected in participants’ urine. In Portugal, the average values detected were higher than those reported in previous studies in European countries, suggesting possible underreporting of exposure or differences in herbicide use patterns.

The presence of glyphosate and AMPA in the urine of North American populations has been detected in both rural and urban settings, across a range of age groups, with concentrations varying depending on factors such as diet, proximity to agricultural areas, and workplace safety practices.

In Mexico [69], the analysis of occupational exposure in agricultural workers revealed alarming levels of estimated intake of glyphosate and AMPA. With relatively high detection limits, extrapolation of the external dose indicated a potential intake of up to 146 mg/kg/day, an extremely high value compared to acceptable limits set by international regulatory agencies such as the EFSA and the EPA. For glyphosate, EFSA establishes an acceptable daily intake (ADI) of 0.5 mg/kg/day [97], while the EPA adopts an even more permissive limit of 1.75 mg/kg/day [98]. Compared to these reference values, the estimated dose represents a higher potential exposure than what is considered safe for chronic use.

In Puerto Rico [70], widespread exposure was detected among pregnant women. Analysis revealed a significant association between urinary levels of glyphosate and AMPA at 26 weeks of gestation and an increased risk of preterm birth. In Canada, two cohorts of pregnant women demonstrated the presence of glyphosate and AMPA in nearly all samples analyzed, with diet identified as the main route of exposure [57]. These data corroborate previous evidence that processed foods and vegetables grown with glyphosate-based herbicides contribute significantly to general population exposure [43].

In the United States, several studies have demonstrated associations between urinary levels of glyphosate and AMPA and adverse health outcomes, particularly in pregnant women and children. Higher concentrations of glyphosate have been associated with reduced gestational length [78,80,99], lower birth weight, and increased risk of neonatal intensive care unit admission [79]. Furthermore, exposure to these compounds during childhood has been associated with an increased risk of metabolic syndrome and liver abnormalities during adolescence [82]. This association was stronger among individuals who lived near agricultural areas during childhood.

On the other hand, in occupational populations such as farmers in the USA [77,87] and Mexico [69], the lack of personal protective measures, such as glove use, was associated with higher urinary glyphosate concentrations. This finding highlights the urgent need for implementation and enforcement of safety protocols in the field, especially among female farmworkers, a group that represents a growing share of the agricultural workforce and may have additional physiological vulnerabilities to pesticide exposure [45,100].

The detection of glyphosate in the urine of participants from African countries, although documented in a limited number of studies, provides relevant evidence of environmental and occupational exposure to this herbicide in the region. In the Democratic Republic of Congo [61], the detection of glyphosate in all participants suggests significant environmental exposure. A multicenter study involving participants from Malaysia, Uganda, and the United Kingdom [68], although not exclusive to the African continent, provides relevant data on the exposure context in Uganda. The results show that higher glyphosate concentrations were associated with behavioral and socioeconomic factors such as lower use of personal protective equipment (PPE), lower educational levels, and longer duration of herbicide use. These associations reinforce the role of occupational vulnerability and social inequality in amplifying health risks arising from pesticide exposure. Specifically, in Uganda, a country highly dependent on the agricultural sector with limited regulations controlling pesticide use, these findings highlight the importance of educational programs, enforcement of good agricultural practices, and access to basic protective measures [101].

In Asian countries such as China and Thailand, widespread pesticide use is evident. In China [59], a broad range of urinary concentrations of glyphosate and AMPA was observed. The concentrations of both compounds showed a correlation with the time-weighted average (TWA), confirming that urinary levels reflect the intensity of occupational exposure.

In Thailand, the three included studies also indicate significant exposure among the rural population. Glyphosate exposure was associated with oxidative stress and impaired lung function among herbicide applicators. Elevated levels of serum malondialdehyde and reduced glutathione were observed, along with worsening respiratory parameters such as forced expiratory volume in the first second (FEV1), the FEV1/FVC ratio, and peak expiratory flow [75]. It was also demonstrated that glyphosate exposure affects the entire rural community population, not just the direct applicators of the product, suggesting that spray drift, soil and water contamination, as well as domestic handling of agricultural products, may represent significant secondary sources of exposure [74,76].

In South America, Brazil stands out as one of the largest consumers of pesticides in the world, especially glyphosate. In 2023 alone, 253,301.95 tons were sold, representing 48% of the total [102]. Despite this widespread use, there are still gaps in monitoring human exposure to this compound by the standard gold method. A study by Panis and colleagues analyzed urine samples from breast cancer patients in southern Brazil by enzyme immuneassay and detected glyphosate residues at concentrations ranging from 0.25 to 2.12 ppb, particularly among those who reported washing work clothes without personal protective equipment. Additionally, women exposed to glyphosate showed a higher risk of developing breast cancer and metastasis [45].

These regional differences in study populations and exposure profiles illustrate the complexity of the problem and the need for specific surveillance and regulatory approaches that consider local contexts. The data suggest that countries with higher pesticide use and more permissive safety limits tend to have higher levels of human exposure, especially among rural workers and socioeconomically vulnerable populations.

### 2.2. Persistent Organic Pollutants (POPs): Dichlorodiphenyltrichloroethane and Hexachlorocyclohexane

#### 2.2.1. Chemical Properties and Associated Health Effects

POPs include non-volatile organic compounds (polychlorinated dibenzo-p-dioxins (PCDDs), dibenzofurans (PCDFs), polychlorinated biphenyls (PCBs), dichlorodiphenyltrichloroethane and its metabolites (DDTs), hexachlorocyclohexane isomers (HCHs), chlordane compounds (CHLs), organochlorine pesticides (OCs), and hexachlorobenzene (HCB)), so named because of their resistance to environmental degradation and high potential for bioaccumulation in wildlife and humans, raising concern due to their cytotoxicity [103].

**Figure 2:**
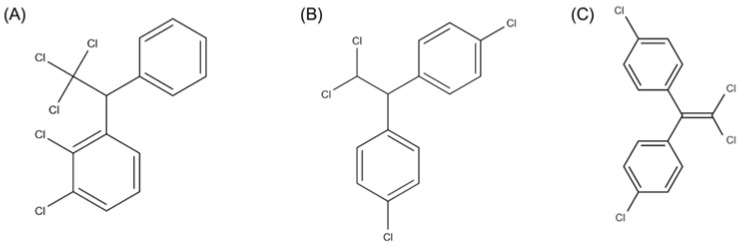
Chemical structure of DDT (**A**), DDD (**B**) and DDE (**C**) [36].

The 2001 Stockholm Convention is an international agreement aimed at protecting human health and the environment from POPs. The stated goal was to reduce or restrict the production and use of DDT (Figure 2) and PCBs, due to their ability to persist in the environment, bioaccumulate, and move over great distances, even to remote regions where they have never been used [104,105,106]. Countries that ratify the convention committed to taking measures to achieve its objectives by eliminating production and use, identifying and monitoring the presence of POPs in their territories, and promoting appropriate waste management to prevent environmental contamination [106].

DDT and HCH are part of the organochlorine pesticides (OCs), a class of synthetic chemicals effective against several insect species and known for their high environmental persistence and highly lipophilic nature, accumulating in food and fatty tissues. This category includes p,p′-dichlorodiphenyltrichloroethane (p,p′-DDT) and its metabolite p,p′-dichlorodiphenyldichloroethylene (p,p′-DDE), β-hexachlorocyclohexane (β-HCH), an isomer of HCH with a longer half-life, and hexachlorobenzenes (HCBs) [107].

In human tissues, bioaccumulation is a concern due to their estrogen-like properties and potential adverse effects in humans [108]. There is considerable evidence of carcinogenic potential in experimental animals, but epidemiological evidence in humans is insufficient, and these compounds are classified by IARC as Group 2B, possibly carcinogenic to humans [45,109].

DDT was once one of the most widely used organochlorine pesticides in agriculture, having been banned in the 1980s, and is currently used primarily to control vector-borne diseases in some developing countries. According to the WHO in 1971, the use of other pesticides, such as malathion or propoxur, as alternatives to DDT would increase the cost of malaria control by approximately 3.4 and 8.5 times, and some countries, such as Africa, would not be able to afford this transition in the coverage of their control programs [110]. Table 2 shows the levels of POPs detected in biological samples from different countries.

The first countries to ban the use of DDT were Hungary, Norway, and Sweden, followed by the United States and West Germany in the 1970s. The United Kingdom and Brazil followed later in the 1980s. Despite this, high levels of this pesticide have been observed in human samples, such as in Portugal in the 2000s, mainly in young women [122]. In some areas of India and Beijing, where the use of these pesticides in agriculture has been restricted, the detected levels were lower (in 2004 and 2019) [111,117], and levels of POPs and DDT found in individuals residing in Delhi were exceptionally high, which is expected in this location because the National Vector-Borne Disease Control Program still recommends indoor residual spraying of homes with DDT for the control of malaria, visceral leishmaniasis, and other vector-borne diseases (2018) [111].

The main reason DDT is still found in the environment and in human samples, even after being banned in many countries, is its status as a persistent organic pollutant (POP). DDT and its byproducts, such as DDE, are chemically stable and slow to degrade, with decomposition taking decades; historical sources, such as contaminated soils and water from sites where DDT was used extensively in the past, continue to release the compound into the environment, and its slow degradation allows it to persist for long periods. As DDT moves through the food chain, its concentration increases at each trophic level, a process known as bioaccumulation or biomagnification, and animals at the top of the food chain, including humans, accumulate much higher concentrations of the compound [122]. Furthermore, DDT is semi-volatile, which allows it to evaporate, travel through the atmosphere, and be deposited in other parts of the globe, including regions where it has never been used, such as the Arctic.

Biological monitoring of DDT exposure can be performed by measuring compounds or their metabolites in blood, serum, plasma, breast milk, and urine samples [3]. However, breast milk has a relatively high amount of fat, making it an ideal matrix for monitoring maternal and infant exposure to these compounds [104]. The detection of different organochlorine pesticides (PCDD/Fs, PCBs) in breast milk samples suggests that the population was exposed to multiple contaminants, which is very common; in China, DDT was predominant in breast milk, with relatively higher levels than in other countries [113]. In Brazil and Colombia, all breast milk samples analyzed showed contamination by DDT and its metabolites, higher than the acceptable daily intake proposed by the WHO [112,114].

In Israel, concentrations of DDT, hexachlorocyclohexanes, and PCBs were above detectable levels in breast milk samples; however, these levels were lower than those reported in European countries [103]. The estimated daily intake of DDT exceeded the tolerable daily intake through breast milk in Tanzania, with a negative correlation with fetal circumference growth [124]. Only Morocco had lower levels of POPs in breast milk compared to other countries, possibly due to its compliance with the requirements of the Stockholm Convention [121].

#### 2.2.2. Regulatory Limits and Restrictions in Different Countries

It is also suspected that in some developing countries, organochlorine insecticides, such as DDT and HCH, are still in use even after the bans, and relatively high levels of these contaminants have been observed in human breast milk [117,126].

The levels detected and reported in studies may reflect past exposures, as many of these compounds have already been banned. These data are not compared with currently permitted intake doses in food or water, as their restricted and controlled use assumes that they would not accumulate in human tissues [127,128], except in countries that still use these pesticides and have WHO oversight in the implementation of malaria control programs, including the safe application of DDT, surveillance of its presence in biological samples, and assessment of its health impacts.

Regarding the health effects of DDT, it has been suggested that exposure is associated with deterioration in semen quality in men [129], early pregnancy loss [130], tumors [131], and diabetes [132]. Chronic effects are of most concern due to their bioaccumulation, and recent evidence indicates a greater risk in women exposed earlier in life [133,134]. Although DDT played an important role in preventing malaria, its risks to human health are significant, especially during infancy, and are associated with higher rates of prematurity and reduced breastfeeding, both important risk factors for infant mortality in developing countries. The WHO is currently reassessing the health risks of DDT, although progress in this assessment has been limited [135].

Biomonitoring programs, such as those conducted by the U.S. Centers for Disease Control and Prevention (CDC), show that DDE levels in the blood and urine of the general population have decreased significantly since the ban, but are still detectable in almost all samples [136]. National and international legislation does not establish “permissible levels” of DDT in the human body, focusing instead on Maximum Residue Limits (MRLs) in food and drinking water to prevent exposure and accumulation. The U.S. Environmental Protection Agency (EPA) and the European Union have strict MRLs for DDT residues in food products such as fruits, vegetables, and meat. The Codex Alimentarius, an international reference, also establishes residue limits for global trade and allows 5 mg/kg of DDT in meat. The maximum permitted concentration in fruits and vegetables in general in the European Union is 0.01 mg/kg, a generic detection limit, reflecting a zero-tolerance policy for the presence of banned pesticides [137]. In the US, an MRL of 0.05 ppm is permitted in food, but most MRLs for DDT have been revoked or reduced to a minimum to reflect its ban on domestic use in the country [51].

In Brazil, the use of DDT has been banned by a series of decrees and laws over the decades. The final and complete ban, for any use, was consolidated in 2009 by Law No. 11,936, which prohibited the production, import, export, storage, marketing, and use of DDT. Thus, DDT is no longer a registered product for use in the country. Regulatory limits for food and the environment are based on the zero-tolerance principle, meaning its presence in food is unacceptable. However, due to its environmental persistence, small amounts of DDT and its metabolites (DDE) can be detected in samples. In these cases, regulations are based on the Limit of Quantification (LQ), which is the lowest concentration of a residue that can be accurately measured through laboratory testing. The Brazilian Health Regulatory Agency (ANVISA), through its Pesticide Residue Analysis Program in Food (PARA), monitors the presence of pesticide residues, including DDT, in plant-based foods sold in the country. If DDT is detected above the LQ, the product is considered to be in violation of the legislation [52].

Hexachlorocyclohexane (HCH) is an organochlorine pesticide that, like DDT, is a POP, formed by a mixture of isomers, the best-known being gamma-HCH (lindane), a potent insecticide. Regulation of HCH is complex because it addresses not only the main substance but also the other isomers (alpha-HCH, beta-HCH) that are byproducts of its production. Beta-HCH is the most persistent and accumulates the most in fatty tissues. Lindane (gamma-HCH), despite having been the main insecticide, is less persistent than beta-HCH. Therefore, regulations often consider total HCH (the sum of all isomers) or specify limits for each of them.

The Stockholm Convention included alpha-HCH, beta-HCH, and lindane (gamma-HCH) in its list of POPs, with the aim of eliminating their production and use. The exception, in some cases, is its use for controlling lice and scabies in humans, but even this use has been gradually phased out. Countries such as the European Union and the United States banned the agricultural use of HCH decades ago, with lindane being discontinued for most uses in the US in 2006 and in the EU in 2002 [138].

Despite the ban, MRLs are established to monitor food contamination, which can still occur due to the persistence of HCH in soil and water. Limits vary by food type and country, reflecting each regulatory agency’s risk assessment. The EU and the US maintain a strict approach, with very low MRLs for HCH and its isomers, typically 0.01 mg/kg, which is the standard limit for unauthorized substances, unless a higher limit is justified. In Brazil, HCH is banned; the tendency is for its limits to be as low as possible, usually below analytical detection limits, to ensure no contamination [139,140].

### 2.3. Carbamates

#### 2.3.1. Chemical Characteristics and General Properties

**Figure 3:**
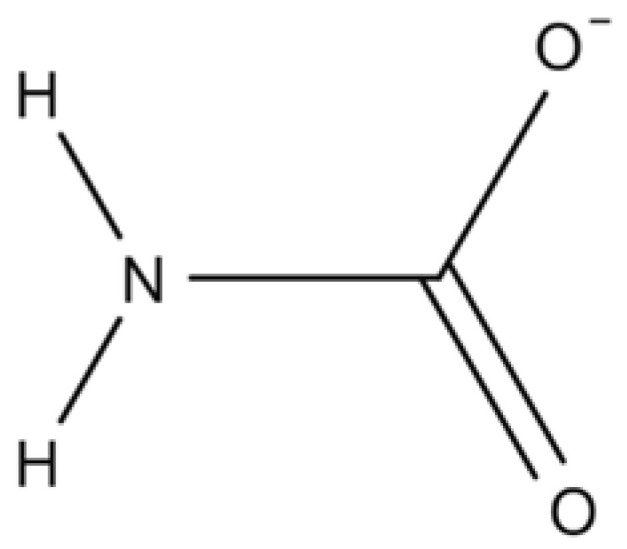
Chemical structure of carbamate [36].

Carbamates constitute a class of substances originating from carbamic acid, a molecule whose inherent instability contrasts with the relatively high stability of its derivatives. Structurally, these compounds feature a characteristic functional group (Figure 3), formed by the substitution of carboxyl and amino groups with organic radicals such as alkyl or aryl chains [141]. These molecules may adopt either cyclic or acyclic conformations. The interaction of the carbamate group with inorganic elements, both metallic and non-metallic, leads to the formation of compounds referred to as inorganic carbamates [142].

Due to their structural and functional versatility, carbamates are employed across multiple fields. In the pharmaceutical sector, they are found in formulations approved by international regulatory agencies, acting as active ingredients or prodrugs in the therapeutic resource for neurological disorders such as myasthenia gravis, alzheimer’s and glaucoma [143]. Within the chemical and agrochemical industries, carbamates assume a central function within the formulation of pest control agents, being particularly valued due to their efficacy and specificity. Furthermore, they are widely used in organic synthesis as protective groups, reactive intermediates, and ligands within the context of combinatorial methodologies [144]. In addition, they serve an important role within the materials industry, where they are incorporated into paint and polyurethane formulations as essential structural elements [142].

Although extensively employed, several carbamates are classified as potentially carcinogenic, prompting regulatory restrictions in various regions, including both the U.S. and the EU [144]. These restrictions derive not only from human health toxicological risks but also on the adverse environmental impacts associated with their use. Among the main concerns are contamination of water sources and soil, atmospheric pollution, and threats to biodiversity in affected ecosystems [145]. At the same time, international regulatory bodies have re-examined health evidence regarding these compounds. The International Agency for Research on Cancer (IARC), for instance, classified carbaryl and aldicarb in Group 3, meaning they are “not classifiable as to their carcinogenicity to humans,” due to insufficient evidence in humans and animals [146,147]. While certain in vitro assays suggest genotoxic potential, such results remain inconclusive for human carcinogenesis [148].

The acute toxicity of carbamate insecticides is typically assessed using the median oral lethal dose (LD_50_) in experiment animals, a standard toxicological parameter inversely proportional to the compound’s toxicity, lower LD_50_ values indicate higher toxic potential. Regulatory authorities such as the World Health Organization (WHO), the U.S. Environmental Protection Agency (EPA), and the European Chemicals Agency (ECHA) have established classification systems based on LD_50_ thresholds to support risk assessment and regulatory decision-making. According to these frameworks, carbamate insecticides can be grouped into three general toxicity categories: high toxicity (LD_50_ ≤ 50 mg/kg), moderate toxicity (LD_50_ between 50–500 mg/kg), and low toxicity (LD_50_ > 500 mg/kg) [149]. In this context, aldicarb stands out as one of the most acutely toxic carbamates, with oral LD_50_ values in rats close to 0.5–1 mg/kg, whereas carbaryl demonstrates comparatively moderate toxicity [150]. Despite the reversibility of acetylcholinesterase inhibition, which reduces the duration of effects relative to organophosphates, highly potent carbamates such as aldicarb and carbofuran can still cause intoxications of comparable severity.

According to the WHO’s Recommended Classification of Pesticides by Hazard, substances are grouped as follows: Class Ia—Extremely hazardous: LD_50_ ≤ 5 mg/kg; Class Ib—Highly hazardous: LD_50_ > 5–50 mg/kg; Class II—Moderately hazardous: LD_50_ > 50–2000 mg/kg; Class III—Slightly hazardous: LD_50_ > 2000 mg/kg [151].

Similarly, the EPA classifies acute oral toxicity for pesticide products into the following categories: Category I—High toxicity: LD_50_ ≤ 50 mg/kg; Category II—Moderate toxicity: LD_50_ > 50–500 mg/kg; Category III—Low toxicity: LD_50_ > 500–5000 mg/kg; Category IV—Very low toxicity: LD_50_ > 5000 mg/kg [152].

#### 2.3.2. Regulatory Residue Limits and Related Health Effects

These classification systems are widely used in toxicological evaluations and regulatory labeling, including the Globally Harmonized System (GHS) adopted by ECHA under EU Regulation (EC) No 1272/2008. The alignment of classification thresholds across these agencies facilitates international harmonization in chemical hazard communication and pesticide management [153]. However, beyond hazard-based categories, risk assessment frameworks in the European Union and other jurisdictions also establish quantitative reference values to better capture chronic and occupational exposures.

For example, the European Food Safety Authority (EFSA) set an acceptable daily intake (ADI) for carbaryl at 0.0075 mg/kg bw/day, while both the acceptable operator exposure level (AOEL) and the acute reference dose (ARfD) were defined at 0.01 mg/kg bw/day [154]. For carbofuran, the ARfD was established at a markedly lower value of 0.00015 mg/kg bw, reflecting its high neurotoxic potential [155]. Aldicarb presents extremely low no-observed-adverse-effect levels (NOAELs), underscoring its high potency [150]. Conversely, propoxur lacks harmonized values in the EU, and maximum residue limits (MRLs) were therefore adjusted to the analytical limit of detection [156].

Epidemiological studies further demonstrate that chronic exposure to carbamates, even at levels consistent with authorized agricultural use, may be associated with neurocognitive deficits in children, respiratory alterations, and possible endocrine disruption [156]. These findings underscore the importance of continuous biomonitoring, strict enforcement of reference values, and progressive restriction of highly hazardous carbamates, especially in low- and middle-income countries where regulatory oversight and enforcement remain limited.

Carbamates inhibit acetylcholinesterase (AChE) through reversible carbamylation of the active site serine residue, leading to a transient accumulation of acetylcholine in the synaptic cleft and the appearance of the cholinergic toxidrome. Unlike organophosphates, this inhibition is not permanent, and the carbamate–AChE complex is unstable and undergoes spontaneous hydrolysis, allowing full enzymatic recovery within 24–48 h without the need for new enzyme synthesis [157,158,159]. This reversibility explains the generally shorter duration and lower severity of carbamate poisoning compared to organophosphates, and also why the use of oximes such as pralidoxime shows inconsistent results in these cases [158,160].

Although carbamates are often described as presenting lower persistence and somewhat less irreversible toxicity compared to organophosphates, this should not be interpreted as an indication of inconsequential health effects. On the contrary, carbamate exposure has been consistently associated with significant acute and chronic outcomes, including severe intoxication and even intentional lethal use in some contexts [158,161,162,163]. Therefore, while mechanistic differences exist between carbamates, organophosphates, and organochlorines, all these pesticide classes pose relevant risks to human health and require careful monitoring and regulation [151,152].

Symptoms observed in acute carbamate poisoning differ according to the absorbed dose, pathway of exposure, as well as individual susceptibility. Symptoms may include muscarinic signs (such as sialorrhea, bradycardia, and bronchospasm), nicotinic signs (such as fasciculations accompanied by reduced muscle strength), along with less common central nervous system manifestations (including agitation, convulsions and coma) [161,162].

Exposure to carbamates may occur acutely or chronically, with absorption possible through multiple routes: dermal, respiratory, ocular, mucosal, or gastrointestinal. Although transdermal absorption is generally limited, it can be significantly enhanced when skin integrity is compromised or when contact occurs with compounds of high lipophilicity. Experimental rodent models indicate that maximal suppression of cholinesterase activity is observed around half an hour following ingestion [161]. Under circumstances of massive exposure, symptom onset can be almost immediate, occurring within as little as five minutes. The latency between exposure and clinical manifestation depends on the amount absorbed and the inherent toxic potential of the specific carbamate compound [164]. Compounds with elevated lipid solubility tend to rapidly partition toward adipose tissue compartments, which may transiently attenuate their initial systemic effects and delay observable toxicodynamics [165].

Carbamates undergo predominant biotransformation within the hepatic system via hydrolytic, hydroxylation, as well as conjugation reactions. Elimination occurs predominantly via the kidneys, with approximately 90% of the compound excreted within a few days [164]. There is still some debate in the literature regarding whether these agents can penetrate the blood–brain barrier and subsequently reach the cerebrospinal fluid [162,166]. Nevertheless, it is observed that while adults typically exhibit limited central neurological involvement, suppression of central nervous system activity is often a prominent clinical feature in pediatric cases [164].

It is important to emphasize that, unlike organophosphates, carbamates do not induce the irreversible enzymatic “aging” process, a phenomenon associated with the permanent phosphorylation of acetylcholinesterase [158]. In contrast, the bond between the carbamate and the enzyme is unstable and dissociates spontaneously within a few hours, generally resulting in a more favorable clinical outcome.

Administration of oximes like pralidoxime for treating intoxication caused by carbamates remains a controversial issue. While their efficacy in treating organophosphate poisoning is well established, outcomes in carbamate intoxication are inconsistent. Table 3 summarizes biomonitoring and clinical findings on human exposure to carbamates across different countries. Reported concentrations range from trace urinary levels in the general population [62] to markedly elevated values in occupational settings and acute poisoning cases [167]. Together, these data highlight the heterogeneous nature of carbamate exposure and reinforce the importance of sensitive analytical methods to support public health monitoring and risk assessment.

The analysis of the data presented in Table 3 highlights the importance of biomonitoring as an essential tool for understanding human exposure to carbamates across different geographic and sociocultural contexts. In China, for example, the use of GC-MS/MS enabled the detection of elevated concentrations of carbofuranphenol in pregnant women and children living in agricultural areas. Across several studies, concentrations detected in urine were consistently associated with adverse developmental outcomes, including reduced birth weight and length, deficits in neurobehavioral performance, lower intelligence quotient, and delayed physical growth [168,169,170,171]. These results demonstrate that even exposures at low levels, when occurring continuously, may exert deleterious effects on vulnerable populations, particularly during critical windows of child development.

In Europe, results from the French national biomonitoring program (Esteban, 2014–2016) revealed low detection frequencies of propoxur, 2-isopropoxyphenol, and carbofuranphenol in samples from adults and children [62]. Although the concentrations observed were lower than those found in populations from highly exposed agricultural regions, the detection of these compounds in the general population indicates the persistence of environmental exposure even in countries with strict regulatory policies. The sensitivity of the analytical method was crucial to uncover these low concentrations, reinforcing the need for continuous monitoring, particularly among more susceptible groups such as children and pregnant women.

In India and Israel, distinct exposure patterns were also identified using sensitive chromatographic methods. Among Indian agricultural workers, elevated levels of carbaryl and propoxur were identified in blood and urine, reflecting mainly occupational exposure [172]. In Israel, urinary quantification of 1-naphthol revealed diffuse exposure to carbaryl, detected in 100% of the samples, although no statistically significant associations were found with dietary factors such as vegetable consumption [148]. These findings highlight that carbamate exposure is not limited to agricultural environments but may also occur in urban contexts through more diffuse environmental routes.

Taken together, the evidence suggests that human exposure to carbamates transcends geographic and socioeconomic boundaries. In countries with high agricultural production, such as China and India, more intense occupational and environmental exposures predominate; in European countries, low but still measurable levels are observed in the general population. These findings underscore that carbamate exposure represents a global public health concern, paralleling the international challenges already highlighted for glyphosate and POPs.

### 2.4. Organophosphate Pesticides

#### 2.4.1. Chemical Properties and Health Effects in Humans

Organophosphate pesticides (OPs) are chemical compounds found in herbicides, pesticides, and insecticides, produced to eliminate and control insects such as fleas, lice, flies, and mosquitoes. They are used in agriculture, livestock, and domestic settings. Due to their faster decomposition and consequently lower persistence in the environment, their use has become a safer and preferable option compared to organochlorines [173,174].

However, due to their high toxicity, there are frequent reports of OP poisoning through ingestion of contaminated food or dust, suicide attempts, and inhalation during occupational exposure to spraying. It acts mainly by preventing the activity of the enzyme acetylcholinesterase (AChE), which is found in the nervous system [174,175]. The toxicity class of OPs is between I and IV via the respiratory or oral route, according to the US-USEPA, and the mechanisms triggered in organisms depend on the type of exposure, quantity of the substance and its chemical structure [176].

**Figure 4:**
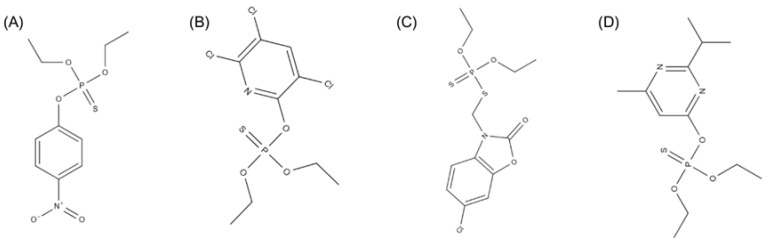
Chemical structure of parathion (**A**), chlorpyrifos (**B**), phosalone (**C**), diazinon (**D**) [36].

The chemical structure of OPs (Figure 4) is essentially composed of a terminal oxygen double-bonded to a phosphoryl group. Attached to the phosphorus atom are two lipophilic groups and a leaving group, usually a halide. These compounds originate from phosphorus esters, commonly derived from thiol or amide derivatives of acids, such as thiophosphoric, and often contain side chains such as thiocyanate, phenoxy and cyanide groups. OPs are resistant to degradation by thermal, photolytic, and chemical processes [176].

When their structure contains an aromatic group such as p-CH_3_-S or p-NO_2_, the electrophilicity of the phosphorus atom increases during metabolic biotransformation in humans, making these compounds more toxic to the organism. However, when the chain length of the lipophilic groups is greater, the central phosphorus atom reduces the electrophilicity, reducing the toxicity of OPs [177]. Most OPs exhibit characteristics such as low water solubility, high volatility, and elevated octanol–water partition coefficients (Kₒw), which reflect their strong lipophilicity and potential for bioaccumulation in biological tissues. Certain OPs, such as diazinon, chlorpyrifos and parathion, have an affinity for fat and can bioaccumulate in human adipose tissue for long periods of time [178].

OPs are active as formulated in pesticides, while others require metabolic activation in the body, particularly those needing conversion to their active oxygen form to exert toxicity. Enzymes such as oxygenases, A-esterases, carboxylesterases, and fluorohydrolases mediate these activation processes through oxidative, reductive, or hydrolytic reactions. For detoxification and elimination, conjugation reactions are necessary, involving enzymes like glutathione S-transferase and amidase [179,180]. The paraoxonase enzyme (PON1) is an A-esterase produced in the liver that breaks the active metabolites of OP compounds that are generated during the first phase of metabolism [180,181,182,183].

Due to their lipophilic characteristics and chemical structure, OPs, like other classes of pesticides, can mimic the action of hormones such as TSH by binding to receptors in the thyroid and activating its signaling pathways. As evidenced in the work carried out in Mexico, with rural works [184], and China [185], with pregnant women. Exposure to these endocrine disruptors contributes to the increase in endocrine disorders in individuals, as well as cardiovascular diseases, neurological problems, bone disorders, and even cancer development [186,187].

OPs, being structural analogs of acetylcholine, covalently bind to the hydroxyl (OH) group of the serine residue at position 203 (Ser203) at the place where the product is transformed by AChE. During the process of adding a phosphate group, OPs phosphorylate the hydroxyl group, rendering the enzyme inactive [188]. AChE is found between motor neurons and muscle fibers and in red blood cells. This inhibition can be reversible or irreversible, as a result of the chemical structure of the OPs [189,190]. These changes can be seen in studies produced in Brazil [191], Greece [192], Thailand [193] and USA [194].

Due to their inhibitory action on AChE, individuals exposed to OPs—either through single or multiple doses—may develop delayed neuropathy, characterized by prolonged uncoordinated and clumsy movements and decreased muscle tone [195,196]. In the USA, it was pointed out that exposed children also demonstrated memory and attention problems [194].

IARC classifies OP insecticides such as malathion and diazinon as probable carcinogens (Group 2A), while dichlorvos, tetrachlorvinphos and parathion are categorized as possible carcinogens (Group 2B) [197]. In Brazil [191] and Portugal [28], analyses of biological samples of an occupationally exposed population revealed changes in genetic material. When metabolized in the body, reactive oxygen species are produced. Thus, in both subchronic and chronic exposure, the main mechanism of OP toxicity is the induction of oxidative stress, defined by increased production of free radicals [198].

The production of free radicals can cause damage to genetic material by inducing oxidation or chain breakage, both directly and indirectly. It may induce mutations in oncogenes and tumor suppressor genes during cell division, potentially leading to carcinogenesis [199,200].

There are also reports in the literature of OPs interfering in sexual reproduction and gametes, described in Spain [201], China [202], and Egypt [203] with female and male rural workers. Oogenesis and spermatogenesis depend on the hypothalamic–pituitary–gonadal axis, which is impaired by the action of OPs, compromising the production of gonadotropins and disrupting the normal regulation of oogenesis and spermatogenesis [204].

Through a literature review and selection of articles meeting established criteria, such as pesticide dosage in human samples, it was observed that studies addressing the effects of OP exposure in populations without acute toxicity symptoms are scarce (Table 4). However, the health impacts of exposure to these substances are well documented by in vitro, in vivo, and observational studies, even when OP levels were not detected in biological samples. Reported health issues include endocrine dysfunction, reproductive problems in both females and males, neurological and motor impairments, genetic damage, mainly by inhibiting the activity of the enzyme AChE, which is responsible for the characteristic symptoms of intoxication. Table 4 presents data from studies analyzing pesticide concentrations in biological samples of individuals in relation to associated health problems.

#### 2.4.2. Regulatory Limits and Different Countries

Because OPs persist in the environment for a relatively short period, they are sometimes considered as alternatives to organochlorines. However, studies on these pesticides worldwide, within the criteria established for this review, are limited. Larger studies have been conducted primarily in China [185,202] and the USA [194,203]. Demonstrating a scarcity of biomonitoring studies of this class worldwide. Most other countries were represented by a single study. The majority of investigations involved exposed workers over 18 years of age, with only one study in the USA including exposed children.

Globally, OPs account for approximately 40% of the total pesticide market. Countries with the highest average annual insecticide use (kg/ha) include Japan (6634), Mexico (2313), Brazil (1163), the USA (0.627), France (0.196), the United Kingdom (0.174), Canada (0.125), Germany (0.124), and India (0.09) [209]. According to the Global Pesticide Market, the compound annual growth rate (CAGR) of OPs between 2018 and 2023 was 5.5%, their use being mainly in developing countries, where the economy is centered on agriculture [210,211].

OP metabolites can be detected in various biological samples, particularly blood and urine. Specific metabolites, as well as dialkyl phosphate metabolites (DAPs), can be identified, making them extremely important for biomonitoring the exposure of rural workers or even those exposed to contaminated food and water [211]. Currently, the most commonly used OPs in agricultural practices are monocrotophos, methylparathion, diazinon, dichlorvos, chlorpyrifos, trichlorfon, and malathime. Some OPs have already been banned in Europe and the USA; however, their extensive use continues in developing countries [212].

In a study, nine metabolites of OPs and pyrethroid pesticides were measured in 322 urine samples collected across eight countries between 2006 and 2014 using HPLC-MS/MS. The highest concentrations were observed in Vietnam (28.9 ng/mL), India (14.2 ng/mL), China (13.6 ng/mL), Korea (12.5 ng/mL), Greece (12.3 ng/mL), Saudi Arabia (11.3 ng/mL), and the USA (7.9 ng/mL) [213].

Even with the increase in biomonitoring studies relating the presence of these metabolites in human samples and their pathological outcomes already observed in adults and children exposed and not exposed to rural areas, as portrayed in this study, and investigation of the amounts of residues present in food and water, there is still negligence on the part of authorities to reduce population exposure [214].

According to EFSA, the NOAEL (No Observed Adverse Effect Level) values of some organophosphates for neurochemical effects are pirimiphos-methyl (0.004–0.4 mg/kg bw), phosmet (0.01–1 mg/kg bw), ethoprofos (0.0004–0.04 mg/kg bw), chlorpyrifos (0.001–0.1 mg/kg bw), dimethoate (0.001–0.1 mg/kg bw), fenamiphos (0.0008 mg/kg bw), methiorcab (0.013–1.3 mg/kg bw) and chlorpyrifos-methyl (0.01–1 mg/kg bw) [215]. The values for the daily intake correspond to malathion (300 μg/kgbw/d), diazinon (5 μg/kgbw/d), parathion (4 μg/kgbw/d), phorate (4 μg/kgbw/d) and dimethoate (2 μg/kgbw/d). It is known, however, that the occupationally exposed population ends up having a higher daily intake of OPs, and the values are in the range of 1.08 to 19.72 μg/kgbw/d [216].

Many countries adopt MRLs for pesticides in food and water as determined by the FAO and WHO. The most commonly used OPs include chlorpyrifos, malathion, dichlorvos, profenofos, dimethoate, and AZM, with MRLs set by the FAO and the WHO at 0–0.01 mg/kg, 0.01 mg/kg, 0.01–7 mg/kg, 0.01–15 mg/kg, 0.01–20 mg/kg, 0.001–4 mg/kg, and 0–0.03 mg/kg, respectively [217]. According to the European Reference Laboratories (EURLs), residual values for diazinon were reported for products of animal origin (LOQ = 0.02 mg/kg) and for general food products (LOQ = 0.05–5 mg/kg) [218]. The EPA and WHO indicate that the maximum permitted levels of organophosphates in water and fish are 30 μg/kg [219]. Analyses on the concentrations of OP residues detected in rivers around the world were developed, and in some of the countries shown in Table 4, values were found, from lowest to highest, respectively, in Greece: 6–30 ng/L^−1^, Spain: 9–97 ng/L^−1^, Mexico: 33–149 ng/L^−1^, Brazil: 535–700 ng/L^−1^, and China: 4–1560 ng/L^−1^ [220].

In Brazil, the estimated average intake of OPs from fruits and vegetables for children and adults was calculated based on FAO and WHO recommendations and analyzed by the Pesticide Residue Analysis Program (PARA) and the National Health Surveillance Agency (ANVISA). Acceptable intake values for OPs generally range from 0.001 to 0.070 g/day [221]. For water intended for human consumption, Brazilian agencies recommend a maximum of 100 μg/L [222]. For China, the average residue threshold in food is 0.05 mg/kg for triazophos and omethoate and 0.1 mg/kg for other OPs investigated. However, residues in rice frequently exceed these limits (0.011–1.756 mg/kg) [223]. A study in Changchun, in northwest China, reported that most foods exceeded permitted levels, with vegetables showing higher contamination than fruits and roots. The associated risk index was 0.448 for adults and 0.343 for children [224]. According to China’s “Agricultural Product Quality and Safety Risk Monitoring Plan,” the geometric mean of OP residues in the general population’s diet ranges from 0.02 to 0.04 μg/kg bw/day [225].

In the USA, the EPA sets permitted residual limits for vegetables, roots, and fruits at 0.001–0.010 ppm for chlorpyrifos, 0.001–0.040 ppm for dichlorvos, 0.001–0.050 ppm for methamidophos, and 0.001–0.020 ppm for omethoate [226]. However, as noted throughout this article, these standards are not particularly stringent and are often difficult to enforce, leaving the population exposed to the adverse effects of OPs [227]. A study by the FDA conducted between 2009 and 2017 reported that chlorpyrifos was among the pesticides most frequently detected in food above the permitted tolerance, with concentrations ranging from 0.06 to 0.08 mg/kg, exceeding the US limit of 0.05 mg/kg [228]. According to NHANES, the most recent urinary analyses for OP metabolite residues in 2018 showed geometric mean values of 2.39 µg/g for DEP, 2.45 µg/g for DMP, 0.298 µg/g for DETP, levels below the detection limit for DEDTP, and 0.481 µg/g for DMDTP [229].

In the European Union, including Spain, Portugal, and Greece, where studies on OP exposure and related human health effects have been conducted, the standards for pesticide application are stricter. Residues of OPs in vegetables ranged from 0.02 to 5 ng/g, with diazinon (0.017 ng/g) and chlorpyrifos (31 ng/g) being the most frequently detected. In fruits, levels generally ranged from 0.006 to 9.32 ng/g, mainly associated with ethoprophos and chlorpyrifos, but none exceeded the maximum permitted residue limits. The risk index was low for both vegetables (3.93) and fruits (4.26), remaining within acceptable limits. These findings highlight the effectiveness of stringent regulatory standards in reducing population exposure to OPs in these countries [230].

In Thailand, urinary levels of OP metabolites in conventional and organic rural workers were analyzed. In this analysis, conventional workers exhibited higher concentrations than organic workers. Another study in the same country examined OP metabolite residues in vegetables used in school meals and correlated them with the presence of these metabolites in children’s urine samples. Analysis of the children’s urine revealed DMDTP at 5258 μmol/mol creatinine and DEP at 2884 μmol/mol creatinine [231].

In Mexico, a study assessed urinary concentrations of OP metabolites in workers with varying levels of pesticide exposure, showing that the metabolite found in the highest concentration was DMTP [232]. In a study conducted in southwestern Mexico, the aim was to analyze the presence of various pesticides in fruits and vegetables classified as highly hazardous, following the criteria established by the FAO/WHO (LD50 < 2 μg/bee). Among these, the presence of chlorpyrifos and methamidophos was detected, exceeding the MRLs published by the country, the latter being identified as highly toxic by the Rotterdam Convention [233].

In Egypt, MRLs established by European Union legislation are generally adopted, with limits of 10 ng/g for chlorpyrifos. However, a study reported that chlorpyrifos levels in buffalo milk exceeded the permitted limit of 0.02 mg/kg [234].

Given the above, it is understood that changes caused by occupational exposure or the ingestion of food and water contaminated by OPs cause numerous types of harm to human health. Therefore, a more rigorous investigation of this class of pesticides is necessary, since their use serves diverse sectors such as agriculture, domestic, industrial, commercial, and governmental use, in addition to being one of the most widely consumed classes of insecticides for these purposes. There is also a disparity in legislation regarding the use of certain OPs among countries, despite extensive scientific evidence regarding their negative effects. In those that adopt prohibitive measures, there is a lack of rigorous investigations into their illegal use in agriculture [235].

## 3. Discussion

The global analysis of exposure to glyphosate and its metabolite AMPA, DDT and other POPs, carbamates, and OPs reveals not only methodological differences in biomonitoring studies but also inequalities in pesticide use regulation, with populations in the Global South frequently exposed to high concentrations of these compounds [236].

In an effort to mitigate the harmful effects of pesticides on human health, the FAO established regulations for pesticide production in 1985. However, many developing countries did not adopt these standards; likewise, the WHO warnings regarding the control of highly toxic pesticides were not followed, leaving populations exposed to their detrimental effects [237].

The role of the agrochemical industry in this scenario is central. The pesticide market generated approximately USD 108.1 billion in 2024, dominated by a few multinational corporations that control around 70% of global sales [238]. Glyphosate, the most widely marketed herbicide worldwide, accounted for about USD 8.99 billion in 2024, and its market value is projected to reach USD 12.44 billion by 2034 [239].

FAOstat data from 2019 to 2025 reveal that some countries in Western Europe, South America, and Oceania, although not the largest absolute consumers (in tons), show high rates of pesticide application per hectare. This pattern reflects the intensification of use in specific agricultural areas, indicating that cultivated soils receive proportionally high chemical loads [240]. Countries with a higher intensity of use per hectare tend to present greater risks of direct and indirect exposure (occupational, environmental, and dietary); however, there is a scarcity of biomonitoring studies in these regions.

The growing expansion of globalization exerts pressure on large-scale food production for both human and animal consumption. Multinational corporations controlling seed and pesticide production have developed genetically modified crops resistant to pesticides, creating a cycle of dependency that compels producers to also use these chemical inputs [241]. This corporate dominance was further highlighted by the divergence between the IARC classification—which places glyphosate, DDT, and OP insecticides such as malathion and diazinon in Group 2A, “probably carcinogenic to humans”—and the broader body of scientific evidence demonstrating carcinogenic effects [44].

Reports produced by international organizations reveal that European Union countries manufacture and export pesticides banned for domestic use to developing countries, exposing a duality in legislation: what is deemed too hazardous for their own populations is not considered harmful enough to prevent export to poorer nations. This dynamic reinforces a trade model that prioritizes economic interests over public health [242].

The global distribution of pesticide exposure reflects not only local agricultural practices but also political and economic dynamics between producing countries, consumers, and regulatory agencies. In countries where pesticide regulations are stricter, exposure tends to be lower and primarily linked to dietary intake.

In Latin America, Asia, and Africa, more intense exposures are observed, particularly among agricultural workers. From a socioeconomic perspective, industrial pressure is disproportionately exerted on rural workers, who represent a vulnerable population. This vulnerability is further aggravated by the fact that populations with lower educational levels tend to use personal protective equipment (PPE) less frequently, thereby increasing occupational exposure, as evidenced by studies conducted in Malaysia, Uganda, and the United Kingdom [68], Mexico [69], and the USA [77,87]. Beyond rural workers, women—often overlooked during the pesticide application cycle—are consistently exposed through secondary contamination, such as when washing clothes of family members involved in spraying [45,84].

From an environmental perspective, the application of pesticides such as glyphosate, POPs, carbamates, and OPs affects soil microbiota and disrupts ecosystems, highlighting that the risks posed by pesticides impact human, animal, and environmental health. Once present in soil and water, these compounds require degradation by specific microorganisms, which are rare and often insufficient to achieve bioremediation, allowing them to persist in the environment for extended periods [176].

This reality demonstrates that pesticide exposure is not limited to the moment of application. In this context, the use of organochlorine pesticides, such as DDT and its derivatives (DDE and DDD), represents one of the greatest public and environmental health dilemmas of the 20th and 21st centuries. Although effective in eradicating pests and controlling disease vectors, their chemical properties of persistence and bioaccumulation pose serious long-term risks. Western countries banned the use of DDT and other organochlorines as early as the 1970s and 1980s; however, before this period, the widespread application of these pesticides resulted in bioaccumulation still observed today, since discussions on the problems caused by these compounds were virtually absent at that time [243]. However, DDT is still used today in some developing countries for the control of vector-borne diseases in endemic areas, mainly due to its low cost [135]. Even with its total ban in some countries, such as Brazil—where regulatory limits in food and the environment are based on the principle of zero tolerance—the presence of DDT and DDE can still be detected in biological samples due to their persistence in the environment, as evidenced by studies conducted in China [113] and in European Union countries such as Luxembourg, Portugal, and Spain [120,122,123]. The levels of these pesticides found in blood, urine, and breast milk samples [117,118] are likely associated with the use of DDT in malaria vector control.

Substances such as carbofuran and aldicarb, belonging to the carbamate group, have been banned by the European Union and the USA due to their high risk; however, countries in Latin America, Africa, and Asia continue to report high levels of agricultural use [154,244]. This class of pesticides shares the same enzymatic target as organophosphates (acetylcholinesterase) but exhibits reversible inhibition, which in some contexts has led to the false perception that they are safer alternatives. However, carbamates do not represent a safe alternative to organophosphates but rather add to the already complex “pesticide cocktail,” increasing the public health risk burden. These effects are demonstrated by studies conducted in rural populations in China [168,169,170,171], France [62], India [167] and Israel [148], where residues were detected in the blood and urine of pregnant women and children, confirming significant environmental and occupational exposure despite the compounds’ rapid metabolism.

Recently, chlorpyrifos, an organophosphate, was banned in the European Union (2019) and the USA (2021) due to the health impacts associated with this class of compounds, including neurological and motor alterations, infertility, and genetic damage, as reported in studies conducted in Spain [206], Portugal [28] and Greece [192] with urine and blood samples collected from children and adults. The Health and Environmental Alliance (HEAL) reported an analysis of pesticide residues in fruits and vegetables, in which chlorpyrifos was among the five most frequently detected compounds exceeding the MRL values [245].

In China, chlorpyrifos was widely used in rice cultivation; however, following studies—such as one conducted with pregnant women linking exposure to thyroid gland alterations [185], along with other evidence of its toxicity to the human body—it was banned by the government in 2018. Nevertheless, due to its persistence in the environment and illegal use in agriculture, its negative effects continue to impact human health, highlighting the ineffectiveness of enforcement measures [246]. Despite being banned in 35 countries, chlorpyrifos continues to be widely used in developing countries such as Brazil, where the economy relies heavily on agriculture and agribusiness, and legislation favors companies and rural producers to increase productivity and profits [247], even in the face of evidence of alterations in cell proliferation and DNA damage reported in studies conducted with rural workers in the country.

Finally, differences in laboratory infrastructure between developed and developing countries impact the assessment of population exposure, leading to an underestimation of the true burden of pesticide exposure and its harmful effects [248]. Countries such as France and the United States maintain robust biomonitoring programs, such as the Esteban Study and the National Health and Nutrition Examination Survey (NHANES), which allow for continuous evaluation of residues in biological samples and support more responsive public policies [55]. Moreover, international initiatives, such as the Codex Alimentarius [217] and Human Biomonitoring for Europe (HBM4EU) [249], aim to harmonize safety parameters and standardize analytical methodologies, strengthening global comparisons and supporting regulatory decisions. In developing countries, such as China and Brazil, the lack of comparable surveillance creates a gap between law and practice, contributing to the continued invisibility of exposure from an epidemiological perspective [250].

In addition to the lack of governmental surveillance to assess the impact of exposure on population health, the high costs and complexity of biomonitoring techniques create difficulties in producing scientific research on this topic in developing countries, such as those in Africa, South Asia, and Central and South America [251]. Population monitoring is extremely relevant, as its results allow for verification of whether regulations limiting pesticide use are being effectively enforced or require adjustments to reduce their harmful effects [252].

Therefore, these points reveal a concerning scenario in which permissive legislation, the lack of systematic monitoring, the absence of remediation mechanisms, and the lack of international regulatory harmonization converge to reinforce risks to human health and environmental balance.

## 4. Concluding Remarks

Human exposure to pesticides remains a global concern, with concentrations in biological matrices ranging from trace levels to values exceeding regulatory limits. Diet, occupational contact, rural life, and socioeconomic conditions are consistently associated with increased exposure. Furthermore, regulatory laxity in countries like Brazil, coupled with insufficient surveillance and enforcement, exacerbates risks compared to the more precautionary European framework. The persistence and bioaccumulation of banned compounds illustrate the irreversibility of environmental and biological contamination, giving pesticide exposure a chronic and transgenerational nature. These vulnerabilities are further exacerbated by socioeconomic inequalities, which disproportionately affect rural workers, women, and children in low- and middle-income countries, where monitoring systems are often limited or nonexistent.

The development of broad-spectrum, nonspecific biomarkers is therefore crucial, along with robust analytical methods and interlaboratory harmonization to ensure data quality and comparability. Without these efforts, exposure patterns will remain poorly characterized, and the lack of standardized reference values will continue to hinder the interpretation of risks and the implementation of preventive interventions.

Finally, existing studies often focus on individual pesticides, while populations are actually exposed to complex mixtures, the so-called “cocktail effect.” The lack of data and methodologies to assess cumulative and synergistic effects represents a significant gap in the understanding of health risks. Across all pesticide classes, children, pregnant women, and agricultural workers emerge as disproportionately affected, reflecting broader socio-environmental inequalities. Furthermore, regulatory fragmentation (from stricter limits in the European Union to more permissive limits in Brazil, the United States, and China) creates an uneven global risk market. Addressing these disparities requires harmonized regulation, coordinated biomonitoring, and integrative risk assessment frameworks that explicitly consider cumulative and synergistic exposures.

## Figures and Tables

**Table 1:** Urinary levels of glyphosate and AMPA detected in samples from different countries.

Country	Sample	Methodology	LOQ	LOD	Outcome (Association)	Reference
Belgium	Urine (*n* = 428)	LC–MS/MS	LOQ GLY/AMPA = 0.1 µg/L	Not reported	Male adolescents living within 2000 m of agricultural areas had increased urinary AMPA levels	[56]
Canada	Urine (*n* = 1880)	UPLC-MS/MS	LOQ GLY = 0.26 μg/L, AMPA = 0.29 μg/L Glufosinate = 0.28 μg/L	LOD GLY = 0.08 μg/L AMPA = 0.09 μg/L Glufosinate = 0.08 μg/L	Because the population is predominantly urban, direct pesticide use and spray drift are unlikely pathways of contamination. Diet is believed to be a potential source of GLY exposure	[57]
Canada	Urine *(n* = 1765)	UPLC-MS/MS	LOQ GLY = 0.26 AMPA = 0.29 μg/L	LOD GLY/AMPA = 0.09 μg/L	No association was identified between urinary concentrations of GLY or AMPA and the risk of preterm birth	[58]
China	Urine (*n* = 134)	GC-MS/MS	Not reported	LOD GLY = 0.02 AMPA = 0.01 mg/L GLY ≤ 0.020–17.202 mg/L (median, 0.292 mg/L); AMPA ≤ 0.010 mg/L–2.730 mg/L (median, 0.068 mg/L)	The urinary concentration of GLY and AMPA of the workers was related to the TWA (time weighted average) value, which reflects the exposure of these workers	[59]
Cyprus	Urine (*n* = 177)	LC-MS/MS	LOQ GLY = 0.19 μg/L AMPA = 0.18 μg/L. GLY = 0.06–3.18 μg/L (mean 0.23 μg/L) AMPA = 0.06–1.44 μg/L (mean 0.24 μg/L)	Not reported	The study identified a significant association between AMPA levels found in the population and oxidative DNA damage	[60]
Democratic Republic of the Congo	Urine (*n* = 15)	LC-MS/MS	LOQ GLY = 80 pg/mL	Not reported	The study indicates exposure to environmental pollutants in the studied population	[61]
France	Urine (*n* = 1299)	UPLC-MS/MS	LOD GLY/AMPA = 0.02 μg/L	LOQ GLY/AMPA = 0.05 μg/L	The study identified higher concentrations of AMPA in children than in adults	[62]
Germany	Urine (*n* = 40)	GC-MS/MS	LOQ GLY = 0.1 μg/L AMPA = 0.1 μg/L. Maximum glyphosate = 2.80 μg/L; Maximum AMPA = 1.88 μg/L	Not reported	Not reported	[63]
Germany	Urine (*n* = 2144)	GC-MS/MS	Not reported	LOD GLY/AMPA = 0.1 µg/L	Not reported	[64]
Germany	Urine (*n* = 301)	LC–MS/MS	LOQ GLY/AMPA = 0.2 µg/L. M GLY = 0.16 µg/L; M AMPA = 0.20 µg/L	LOD GLY = 0.05 µg/L, LOD AMPA = 0.09 µg/L	The study identified positive correlations between urinary glyphosate excretion and legume consumption, as well as between urinary AMPA excretion and mushroom consumption	[65]
Ireland	Urine (*n* = 50)	LC-MS/MS	Not reported	LOD GLY = 0.5 µg/L. Median = 0.87 µg/L	Results suggest environmental exposure to GLY	[66]
Italy	Urine (*n* = 17)	UPLC-MS/MS	LOQ GLY = 0.1 μg/L AMPA = 0.5 μg/L. Median GLY = 2.30 μg/L	Not reported	Reports the absorption of xenobiotic by vineyard workers after application of GLY	[67]
Malaysia, Uganda and United Kingdom	Urine (*n* = 271)	LC-MS/MS	LOQ GLY/AMPA = 0.5 μg/L	Not reported	Higher GLY concentrations were associated with lower use of personal protective equipment, lower education levels, and longer duration of pesticide use	[68]
Mexico	Urine (*n* = 30)	HPLC	Not reported	LOD GLY = 5.0 AMPA = 15 μg/L	The study identifies, through the calculation of the external dose, that agricultural workers ingest up to 146 mg/kg/day of GLY	[69]
Puerto Rico	Urine (*n* = 247)	GC-MS/MS	Not reported	LOD GLY/AMPA = 0.20 μg/L	Study identifies associations between urinary GLY and AMPA concentrations and increased likelihood of preterm birth	[70]
Portugal	Urine (*n* = 79)	GC-MS/MS	LOQ GLY = 0.05 μg/L	LOD GLY = 0.02 AMPA = 0.013 μg/L	GLY and AMPA were detected in the study population, in both rounds of testing and in concentrations above the values of previous studies carried out in European countries	[71]
Slovenia	Urine (*n* = 246)	GC-MS/MS	LOQ GLY/AMPA = 0.1 µg/L	Not reported	Study shows low exposure to GLY and AMPA	[72]
Spain	Urine (*n* = 97)	LC-MS/MS	LOQ GLY/AMPA = 0.1 µg/L	Not reported	GLY concentration was related to the consumption of eggs and fruits in the 72 h preceding urine collection	[73]
Thailand	Urine (*n* = 124)	HPLC	Highest concentration = 2.92 ng/ml	LOD GLY = 0.5 μg/L	Exposure to GLY affects the entire population living in the agricultural community	[74]
Thailand	Urine (*n* = 180)	LC-MS/MS	LOQ GLY = 5 g/L	LOD GLY = 2.5 g/L	Workers applying GLY presented greater oxidative stress and worsening of lung function	[75]
Thailand	Urine (*n* = 15)	HPLC	LOQ GLY = 0.4 μg/L. M = 19.42 μg/L. Maximum level = 57.49 μg/L	LOD GLY = 0.1 μg/L	Workers are at significant risk of exposure to GLY	[76]
USA	Urine (*n* = 175)	LC-MS/MS	LOD GLY 1 ppb	LOD GLY 1 ppb Glyphosate concentrations ≤ 1–233 ppb	Farmers who did not use personal protective equipment, such as rubber gloves, had higher urinary GLY concentrations than other farmers	[77]
USA	Urine (*n* = 71)	LC-MS/MS	LOQ GLY = 0.5 ng/mL	LOD GLY = 0.1 ng/mL	Shortened gestational length was associated with high urinary GLY levels	[78]
USA	Urine (*n* = 187)	HPLC	LOQ GLY = 0.5 ng/mL	LOD GLY = 0.1 ng/mL	Elevated maternal GLY levels in the first trimester of pregnancy were associated with lower body weight percentiles (BWT) and increased risk of NICU admission	[79]
USA	Urine (*n* = 368)	IC-MS/MS	Not reported	LOD GLY = 0.2 µg/L	Study suggests that GLY exposure may be associated with oxidative stress	[80]
USA	Urine (*n* = 52)	LC-MS/MS	LOQ GLY = 0.03 ng/mL	LOD GLY = 0.03 ng/mL	Urinary GLY concentration was associated with increased odds of preterm birth (adjusted for urine specific gravity and maternal race)	[81]
USA	Urine (*n* = 480)	UPLC-MS/MS	Not reported	LOD GLY = 0.08 µg/L AMPA = 0.09 µg/L	Detection of AMPA in urine in children at 5 years of age was associated with elevated transaminases and metabolic syndrome. At 14 years of age, it was associated with metabolic syndrome. Overall, increased urinary AMPA during childhood was associated with an increased risk of elevated liver transaminases and metabolic syndrome. Individuals who lived near agricultural areas during early childhood were also associated with metabolic syndrome at age 18	[82]
USA	Urine (*n* = 305)	MS	LOQ GLY = 0.041 AMPA = 0.040 ng/mL	LOD GLY = 0.014 AMPA = 0.013 ng/mL	The study suggests that grains are the largest dietary source of GLY, while AMPA is found in soybeans, wine and fast food	[83]
USA	Urine (*n* = 62)	UPLC-MS/MS	Not reported	LOD GLY/glufosinate = 0.08 μg/L AMPA = 0.09 μg/L	Women represent a growing proportion of agricultural workers and may be uniquely vulnerable to pesticides	[84]
USA	Urine (*n* = 40)	IC-MS/MS	Not reported	LOD GLY = 0.1 μg/L	The study suggests that people living near agricultural areas suffer greater exposure to GLY	[85]
USA	Urine (*n* = 2132)	IC-MS/MS	Not reported	LOD GLY = 0.2 μg/L. Concentrations = 0.141–8.210 μg/L	Urinary GLY has been linked to decreased muscle strength and increased prevalence of physical function limitations in middle-aged and older adults	[86]
USA	Urine (*n* = 368)	MS	Not reported	LOD GLY = 0.2 µg/L	Farmers who did not wear rubber gloves when handling the pesticide had higher urinary GLY concentrations	[87]
USA	Urine (*n* = 2745)	IC-MS/MS	Not reported	LOD GLY = 0.2 μg/L	Increased urinary GLY concentrations were associated with a 46% increase in diabetes prevalence (after adjustment for covariates)	[88]
USA	Urine (*n* = 5224)	IC-MS/MS	Not reported	LOD GLY 0.2 ng/ml	Urinary GLY concentration was associated with a statistical score that was developed to predict the presence of metabolic syndrome	[89]
USA	Urine (*n* = 2842)	IC-MS/MS	GLY concentration 0.31 ng/mL	Not reported	Exposure to GLY may pose a risk to cardiovascular health, with elevated serum insulin levels being a key mediator in this process	[90]
USA	Urine (*n* = 2094)	IC-MS/MS	Not reported	LOD GLY 0.2 µg/L. M = 0.304 μg/g creatinine	Urinary GLY levels have been associated, in a dose-dependent manner, with glycemic dyshomeostasis, especially in individuals with overweight/obesity or central obesity	[91]
USA	Urine (*n* = 1689)	IC-MS/MS	Not reported	LOD GLY 0.2 ng/mL	GLY levels in urine correlate with the severity of arthritis, specifically its osteoarthritis subtype	[92]

Abbreviations: GLY: glyphosate, AMPA: aminomethylphosphonic acid, c, LOQ: Limit of Quantitation, USA: United States of America, GC-MS/MS: gas chromatography–tandem mass spectrometry, LC-MS/MS: liquid chromatography–tandem mass spectrometry, UPLC-MS/MS: high-performance liquid chromatography–tandem mass spectrometry, HPLC: high-performance liquid chromatography, M: median, MS: mass spectrometry, IC-MS/MS: ion chromatography–tandem mass spectrometry, GC-MS/MS: gas chromatography-electrospray ionization–mass spectrometry.

**Table 2:** Levels of POPs detected in biological samples from different countries.

Country	Sample	Methodology	LOD	LOQ	Outcome (Association)	Reference
Beijing	Blood (*n* = 100)	GC/MS	LOD (0.4 and 21.6 ng/mL)	LOQs = 1.4 and 71.9 ng/mL. LOQ p,p′-DDD = 0–400.7 ng/g of lipid LOQ p,p′-DDT (3.0–2534.1 ng/g of lipid) LOQ total DDT (25.4–9361.9)	The blood of the analyzed individuals indicated low exposure of the general population of Beijing to several POPs compared to other countries.	[111]
Brazil	Breast milk (*n* = 69)	GC	LOD p,p′-DDE (0.0040 ng/mL) LOD p,p′-DDD (0.0340 ng/mL) LOD p,p′-DDT (0.0040 ng/mL)	LOQ not reported	In a study in Brazil, all breast milk samples were contaminated with DDT and its metabolites and 8.7% of the estimated daily intake (EDI), in terms of total DDT, a value higher than the acceptable daily intake proposed by the WHO.	[112]
China	Breast milk (*n* = 40)	Gel permeation chromatography	LOD p,p′-DDT = 12–380 ng/g LOD p,p′-DDD ≤ 0.51–28 ng/g LOD Σ DDTs = 780–5400 ng/g	LOQ not reported	The most common contaminant found in human breast milk was DDTs, with higher levels than in other countries. An estimate of daily DDT intake by infants from human breast milk was made, and breast milk from Dalian had higher concentrations of DDTs compared to samples from Shenyang, suggesting that infants from Dalian may be at higher risk from these contaminants.	[113]
Colombia	Breast milk (*n* = 60)	GC	LOD PBDEs: 2 a 42 pg/g LOD PCBs: 16 a 99 pg/g LOD OCPs: 8.55 a 89.2 pg/g	LOQ PBDE: 2.5–54.7 pg/g LOQ PCBs: 39–271 pg/g LOQ OCPs: 20.4–259 pg/g	DDT concentrations were found in breast milk samples from women in Colombia at levels comparable to those found in other countries, such as Brazil, Uruguay, Chile and Asian countries.	[114]
Delhi	Blood (*n* = 50)	GC	LOD OCPs: 4 pg/mL	LOQ not reported	The levels of β-HCG found in the blood of the study subjects were associated with the pathogenesis of metabolic syndrome.	[115]
France	Blood (*n* = 386)	GC	HCB = 22.8 ng/g α- HCH = 0.74 ng/g ß-HCH = 27.0 ng/g DDT = 3.8 ng/g DDE = 104.6 ng/g LOD not reported	LOQ not reported	Serum levels of OC pesticides in French adults were higher (except for DDT and DDE) than levels found in American, Canadian, and German populations, and lower than or similar to levels found in other European countries.	[116]
India	Blood (*n* = 18)	GC	M = 32.61 µg/L LOD not reported	LOQ not reported	The levels of total DDT and total HCG found in the blood of the individuals were lower than previous findings from India, which may be due to the restriction on the use of these pesticides in agriculture.	[117]
India	Blood (*n* = 193)	GC/MS	LOD not reported	LOQ p,p-DDE = 330 (273–399) ng/g LOQ p,p-DDE = 579 (521–643) ng/g	The levels of p,p′-DDE in the blood of individuals from Delhi were exceptionally high, which is expected in this locality because the National Vector-borne Disease Control Programme still recommends indoor residual spraying of homes with DDT for the control of malaria, visceral leishmaniasis and other vector-borne diseases.	[118]
Israel	Breast milk (*n* = 52)	GC/MS	M DDT = 168 ng/g M DDE = 147 ng/g M p′p DDT 4.3 ng/g LOD not reported	LOQ not reported	Compounds of DDT, hexachlorocyclohexanes and PCBs were above detectable levels in breast milk samples from Israeli women. Although considered high, the levels were still lower than those reported in European countries included in the WHO/UNEP study.	[103]
Korea	Blood (*n* = 1295)	HRGC/HRMS	p,p′-DDE = M = 128.47 ng/g of lipid LOD not reported	LOQ not reported	More than 60% of the blood samples analyzed had HCB, p,p′-DDT, and p,p′-DDE at concentrations lower than or similar to those reported in Korean nationwide biomonitoring surveys.	[119]
Luxembourg	Hair analysis (*n* = 497)	GC/MS	M γ-HCH = 0.37 pg/mg M lindano = 0.15 pg/mg M HCB = 14.1 pg/mg LOD not reported	LOQ not reported	In unusual hair samples, 17 chemicals were found in concentrations of these chemicals comparable to those found in studies conducted in France and China. The main compounds found were γ-HCH, lindane and HCB.	[120]
Morocco	Blood (*n* = 59)	GC/MS	∑DDT = 237.9 ng/g. LOD not reported	LOQ not reported	Low levels of POPs were observed in human milk from Moroccan women compared to those from other countries, reflecting the effectiveness of compliance with the Stockholm Convention requirements in the country.	[121]
Portugal	Blood (*n* = 203)	GC	LOD not reported	LOQ p,p′ DDE = 43.5–390.25 µg/L	Blood levels of p,p′DDE ranged from undetected to 390.5 µg/L in urban samples, and lower levels (undetected to 43.5 µg/L and 171.2 µg/L) in rural samples. Portugal presented in this study one of the highest levels of contamination when compared to levels found in Europe, Asia and America.	[122]
Saudi Arabia	Umbilical cord /maternal serum (*n* = 1578)	GC	Umbilical cord and maternal: LOD p,p′-DDE = 0.085 μg/L LOD p,p′-DDD = 0.186 μg/L LOD p,p′-DDT = 0.202 μg/L, Placental tissues: LOD p,p DDE = 0.043 μg/kg dry weight LOD p,p-DDD = 0.093 μg/kg dry weight LOD p,p-DDT = 0.1 μg/kg dry weight	LOQ not reported	p,p′-DDE and p,p′-DDT were found in the umbilical cord and maternal serum, and placental tissues. Based on these findings, the authors report that intrauterine exposure to DDT may have caused a reduction in fetal head circumference, crown-to-heel length, and birth weight.	[123]
Spain	Blood (*n* = 953)	GC	M p,p′-DDE = 822 ng/g M β-HCH = 167 ng/g M HCB = 379 ng/g MLOD not reported	LOQ not reported	Relatively high levels of OCPs were found in the blood of the subjects in this study, mainly in those involved in agricultural practices. Serum HCB levels were particularly high in one of the regions due to industrial use. In general, serum β-HCH and HCB levels were found to be substantially higher than in most Western countries.	[105]
Tanzania	Breast milk (*n* = 95)	GC	LOD p,p′-DDE = 24–2400 ng/g LOD p,p′-DDT = undetected/133 ng/g	LOQ not reported	The estimated daily intake of all DDTs (parent compound and metabolites) exceeded the tolerable daily intake in this study with breast milk. Decreased fetal head circumference was associated with high levels of p,p′-DDE in female infants, suggesting that exposure to OCs during pregnancy may influence fetal growth.	[124]
Thailand	Blood (*n* = 97)	GC	LOD p′, p′-DDE = 1325.1–12,683.7 ng/g	LOQ not reported	The levels of p′,p′-DDE found in the blood of the research subjects were relatively higher when compared to other studies.	[125]

Abbreviations: DDTs: dichlorodiphenyltrichloroethane and its metabolites; ∑DDT (sum of o,p′-DDD, p,p′-DDD, o,p′-DDE, p,p′-DDE, o,p′-DDT and p,p′-DDT); p,p′- DDT: 1,1,1-trichloro-2,2-bis-(p-chlorophenyl)ethane); p,p′DDE: 1, 1-dichloro-2, 2-bis (p-chlorophenyl) ethylene; p,p′-DDD: 1, 1-dichloro-2, 2- bis (p-chlorophenyl) ethane; s: chlordane compounds; OCs or OCPs: organochlorine pesticides; GC: gas chromatography, HCB: hexachlorobenzene; HRGC: high-resolution gas chromatography, HRMS: high-resolution mass spectrometry α-HCH: alpha-Hexachlorocyclohexane; β-HCH: beta-Hexachlorocyclohexane; γ-HCH: gamma-Hexachlorocyclohexane or lindane; PCBs: polychlorinated biphenyls; MS: mass spectrometry, M: average, LOD: Limit of Detection, LOQ: Limit of Quantitation.

**Table 3:** Carbamate exposure: analysis of biological samples and clinical implications.

Country	Sample	Methodology	Levels Found	LOD	LOQ	Outcome (Association)	Reference
China	Urine (pregnant women, *n* ≈ 1100; Sheyang Birth Cohort)	GC-MS/MS	Carbofuranphenol detected in 96.6% of samples; GM ≈ 0.81 µg/L (IQR: 0.29–2.07 µg/L); values up to 395.4 µg/L	0.01 µg/L	0.05 µg/L	Urinary concentrations associated with lower birth weight and reduced length of newborns	[168]
China	Urine (children age 3, *n* ≈ 300; SMBCS Cohort)	GC-MS/MS	Carbofuranphenol GM ≈ 0.70 µg/L; P95 ≈ 5.6 µg/L	0.01 µg/L	0.05 µg/L	Carbamate exposure associated with worse neurobehavioral performance in young children	[169]
China	Urine (children age 7, *n* ≈ 400; SMBCS Cohort)	GC-MS/MS	Carbofuranphenol median ≈ 0.56 µg/L; P95 ≈ 4.9 µg/L	0.01 µg/L	0.05 µg/L	Early exposure to carbamates associated with reduced IQ at age 7	[170]
China	Urine (children age 7, *n* ≈ 420; SMBCS Cohort)	GC-MS/MS	Carbofuranphenol median ≈ 0.62 µg/L; range 0.05–9.4 µg/L	0.01 µg/L	0.05 µg/L	Carbamate exposure associated with delayed physical development (lower height and weight)	[171]
France	Urine (children, *n* = 500)	UPLC-MS/MS	Propoxur (P95) = 0.05 µg/L; 2-isopropoxyphenol (P95) = 0.30 µg/L; carbofuranphenol < LOQ (máx = 12.2 µg/L)	Propoxur/2-IPP = 0.02 µg/L; carbofuranphenol = 1 µg/L	Propoxur/2-IPP = 0.05 µg/L; carbofuranphenol = 5 µg/L	Low frequency of quantification in the general population	[62]
France	Urine (adults, *n* = 899)	UPLC-MS/MS	Propoxur (P95) = 0.13 µg/L; 2-isopropoxyphenol (P95) = 0.19 µg/L; carbofuranphenol < LOQ (máx = 13.4 µg/L)	Propoxur/2-IPP = 0.02 µg/L; carbofuranphenol = 1 µg/L	Propoxur/2-IPP = 0.05 µg/L; carbofuranphenol = 5 µg/L	Low levels in the general population; quantification in 1.7–8.5% of adults	[62]
India	Blood/urine (*n* = 42)	LC-MS/MS	Carbaryl/Propoxur = 0.3 to 2.5 µg/L	Not reported	LOQ = 0.1 µg/L	Elevated carbamate levels were observed among agricultural workers, suggesting occupational exposure	[167]
Israel	Urine (***n*** = 42)	GC-MS	1-naphthol = detected in 100% of urine samples IPPX = detectability less than 20%.	Not reported	Not reported	Higher 1-naphthol levels compared to other populations reported in the literature- No statistically significant association with vegetarian diet- IPPX with levels too low for association analyses	[148]

Abbreviations: LOD: Limit of Detection; LOQ: Limit of Quantification; GC-MS: gas chromatography–mass spectrometry; GC-MS/MS: gas chromatography coupled with tandem mass spectrometry; UPLC-MS/MS: ultra-performance liquid chromatography coupled with tandem mass spectrometry; LC-MS/MS: liquid chromatography coupled with tandem mass spectrometry.

**Table 4:** Mean levels of organophosphate pesticides detected in biological samples from different countries.

Country	Sample	Metodology	LOQ	LOD	Outcome (Association)	Reference
Brazil	Urine (*n* = 188)	GC/MS	LOQ DETP = 0.078 μgL^−1^ LOQDEDTP = 0.0431 μgL^−1^	LOD DETP = 0.023 μgL^−1^ LOD DEDTP = 0.0129 μgL^−1^	Decrease in AChE and DNA damage, problems in cell division, changes in proliferative potential, and cell death	[191]
China	Serum (*n* = 325)	GC/MS	LOQ DMTP = 2.27 μg/L LOQ DEP = 11.20 μg/L LOQ DETP = 1.99 μg/L	LOD DMP = 9.81 μg/L LOD DMTP = 0.79 μg/L LOD DEP = 5.00 μg/L LOD DETP = 0.78 μg/L LOQ DMP = 21.40 μg/L	Association with FT4 and TSH. Changes in thyroid function in pregnant women	[185]
China	Semen/ Urine (*n* = 75)	GC	Not reported	LOD p-nitrophenol = 0.018 mg/L	When comparing exposed and unexposed works, the analysis of the sample of the first spermatozoa showed worse characteristics with decreased quantity and motility	[202]
Egypt	Follicular fluid (*n* = 300)	GC/MS	Not reported	LOD Chlorpyrifos = 0.098 μg/L LOD Diazinon = 0.055 μg/L LOD Malathion = 0.024 μg/L LOD Pretilachlor = 0.003 μg/L LOD DDT = 0.005 μg/L LOD Lindane = 0.083 μg/L	Decrease in the oocytes retrieved. High concentrations associated with lower implantation	[205]
Greece	Blood/hair (*n* = 1)	GC/MS	LOQ DMP = 1289.4 pg/mg LOQ DEP = 709.4 pg/mg, LOQ opDDE = 484.0 pg/mg, LOQ ppDDE = 526.6 pg/mg, LOQ opDDD = 448.4 pg/mg, LOQ ppDDD + opDDT = 259.9 LOQ pg/mg, ppDDT = 573.7 pg/mg	Not reported	Exposure association with motor neuron disease	[192]
Mexico	Urine/ blood (*n* = 148)	GC-liquid/GC	Not reported	LOD ΣDMP = 1.45 μmol/g creatinine LOD ΣDEP = 0.31 μmol/g creatinine LOD ΣDAP = 1.99 μmol/g creatinine LOD p,p-DDE = 6.22 ng/mL	OPs alter the amounts of thyroid hormone, especially in those with PON1 192RR polymorphism or even decreased PON1 enzyme activity	[184]
Portugal	Urine (*n* = 85)	colorimetric kit	Not reported	LOD 2.23 μg/mmol	Increase in DNA strand breaks and alterations in the percentage of B lymphocytes	[28]
Spain	Urine (*n* = 116)	GC/MS	LOQ DEDTP = 0.01 µg/L LOQ DAP = 0.1 µg/L	LOD DMP = 1.3 µg/L LOD DMTP = 1.0 µg/L LOD DMDTP = 0.08 µg/L LOD DEP = 2.6 µg/L LOD DETP = 0.94 µg/L LOD DEDTP = 0.05 µg/L LOD ∑DAP = 75.5 nmol/L LOD ∑DAP = 100 mg/L	OPs have been associated with male infertility, as they affect the biosynthesis of hormones responsible for reproduction, consequently reducing the number of sperm, as well their mobility	[206]
Thailand	Urine/ Blood (*n* = 150)	GC	LOQ DETP = 1 μg/L LOQ DMP = 25 μg/L	LOD DMP = 5.0 μg/g LOD DMTP = 0.4 μg/g LOD DMDTP = 0.36 μg/g LOD DEP = 0.51 μg/g LOD DEDTP = 0.4 μg/g LOD DETP = 0.26 μg/g LOD DETP = 0.1 μg/L LOD DMP = 2.5 μg/L	Muscle weakness in exposed individuals	[193]
USA	Urine (*n* = 26)	GC/MS	LOQ AZM = 5.30 μg/g LOQ DMP = 4.0 ng/mL LOQ DEP = 2.0 ng/mL LOQ DMTP = 2.2 ng/mL LOQ DMDTP = 1.6 ng/mL LOQ DETP = 1.6 ng/Ml	LOD DMP/DEP/DMTP/DMDTP/DETP = 0.01 μg/gm LOD AZM = 0.10 μg/gm	Neurological and behavioral assessment using questionnaires applied to works exposed to OPs indicated worse performance compared to those who did not work with crops, therefore not exposed	[207]
USA	Urine (*n* = 652)	GC/FPD	Not reported	LOD MP ≥ 100 ppb	The assessment test applied to verify neurological and motor performance showed that children exposed to OPs have the worst results	[208]

Abbreviations: AZM: Azinphos-methyl, AChE: Acetylcholinesterase, DAP: ialkylphosphate, DEDTP: Diethyldithiophosphate, DEP: Diethylphosphate, DETP: Diethylthiophosphate, DMDTP: Dimethyldithiophosphate, DMP: Dimethylphosphate, DMTP: Dimethylthiophosphate, FT4: free T4, GC: gas chromatography, MS: mass spectrometry, LOD: Limit of Detection, LOQ: Limit of Quantification MP: Methyl parathion, OP: Organophosphate, p,p′-DDE: P,p′-dichlorodiphenyldichloroethene, PON1: Paraoxonase-1 enzyme, USA: United States of America.

## Data Availability

No new data were created or analyzed in this study. Data sharing is not applicable to this article.

## Supplementary Materials

The following supporting information can be downloaded at: https://www.mdpi.com/article/10.3390/jox15060187/s1. We added the index to the supplementary material.
