# Supplementary material for: Global Evidence on Monitoring Human Pesticide Exposure
Source: J Xenobiot. 2025 Nov 7;15(6):187. doi: 10.3390/jox15060187 (PMC12641724; doi:10.3390/jox15060187)
Supplement: Supplementary file 1 [file jox-15-00187-s001.zip › jox-3757471-supplementary.pdf]

Journal of Xenobiotics

MDPI

Supplementary Material: Global Evidence on Monitoring Human Pesticide Exposure

Tatiane Renata Fagundes, Carolina Coradi, Beatriz Geovana Leite Vacario, Juliana Maria Bitencourt de Morais Valentim and Carolina Panis

Index

1. Introduction ... 1

2. Pesticide Levels in Human Samples: Primary Matrices, Methods, and Target Analytes ... 3

2.1.Glyphosate-AMPA ... 6
2.1.1 Chemical composition and general properties ... 6
2.1.2 Regulatory limits in different countries and health effects in humans ... 7
2.2. Persistent Organic Pollutants (POPs): Dichlorodiphenyltrichloroethane and Hexachlorocyclohexane ... 14
2.2.1 Chemical properties and associated health effects ... 14
2.2.2 Regulatory limits and restrictions in different countries ... 19
2.3.Carbamates ... 20
2.3.1 Chemical characteristics and general properties ... 20
2.3.2 Regulatory residue limits and related health effects ... 21
2.4.Organophosphate Pesticides ... 25
2.4.1 Chemical properties and health Effects in Humans ... 25
2.4.2 Regulatory limits and different countries ... 29
3.Discussion ... 31
4. Concluding Remarks ... 33
References ... 34
